# Supplementary material for: An approach to linking education, social care and electronic health records for children and young people in South London: a linkage study of child and adolescent mental health service data
Source: BMJ Open. 2019 Jan 29;9(1):e024355. doi: 10.1136/bmjopen-2018-024355 (PMC6352796; doi:10.1136/bmjopen-2018-024355)
Supplement: Supplementary file 1 [file bmjopen-2018-024355supp001.pdf]

SUPPLEMENTARY MATERIAL

Title: An approach to linking education, social care and electronic health records for children and young people attending mental health services

Johnny Downs, Tamsin Ford, Robert Stewart, Hitesh Shetty, Ryan Little, Amelia Jewell, Matthew Broadbent, Jessica Deighton, Tarek Mostafa, Ruth Gilbert, Matthew Hotopf and Richard Hayes

* Corresponding author contact information:

Dr Johnny Downs, Department of Child and Adolescent Psychiatry, IOPPN Biomedical Research Centre Nucleus, Ground Floor Mapother House PO BOX 92, King's College London, De Crespigny Park, Denmark Hill, London SE5 8AF. Tel: +44 (0)20 3228 8553. Email: johnny.downs@kcl.ac.uk

Supplementary report on providing a linked health and educational data resource: achieving the ethical, governance and legal approvals

Initiating the discussion on the purpose and process of the linkage between public sector data controllers

We first approached the Department for Education directly who held nationally collected education data via termly school submissions to the National Pupil Database.[1] We planned a linkage with national data, as opposed to regional data sources held by the local education authorities, to prevent clinical sample attrition. We expected a considerable proportion of children and young people receiving SLaM treatment would reside outside the SLaM Catchment area or potentially move outside the catchment after treatment. In addition, the Department for Education had relatively transparent systems, and a dedicated office, for managing requests for educational data extracts, through their National Pupil Database Team. Once Research Governance approval was granted by the SLaM Caldicott Guardian Committee and the DfE's Data Management Advisory Panel, we prepared an application to the Health Research Authority Confidentiality Advisory Group (HRA CAG).[2] The HRA CAG have the authority to provide recommendations on behalf of the Secretary of State for Health to permit the linkage of NHS data without individual patient consent for the purposes of research, if it meets the criteria within section 251 of the NHS Act 2006. The main purpose of our application was to examine and

estimate the effects of clinically recognised, mental health disorder and treatment on educational outcomes.

The HRA CAG rejected the first application, as the research activity proposed did not demonstrate sufficient medical purpose and public benefit to meet the s251 requirements. It was highlighted by the HRA CAG that support under current regulations could only be provided where potential public benefit were sufficiently defined.[3] In particular, it was noted that in order to satisfy one of the conditions in schedule 3 of the Data Protection Act (32) (required to process sensitive personal data including data relating to an individual's physical or mental health) a medical purpose would also need to be specified; education outcomes in themselves would not suffice as a medical outcome. A second issue, was the lack of consideration of a practicable alternative to the use of confidential patient information without consent.

The HRA CAG also queried whether we had considered if the Health and Social Care Information Centre (HSCIC, now NHS Digital)[4] could carry out the linkages on the applicant's behalf using their Trusted Data Linkage Service. The CAG advised that this route would negate the requirement for SLaM to disclose confidential patient information to the DfE, and minimise the disclosure of patient information. A final major issue related to the governance arrangements in place around the processing of patient data by the DfE. We hadn't provided sufficient information around retention periods, access arrangements and the extent of identifiable data requested.

Defining 'medical purpose' and public benefit when seeking s251 support

To prepare for resubmission, we examined the issues identified by the HRA CAG. The initial application took a broad interpretation of 'medical purpose.' Given our clinical experience working in CAMHS, and the time CAMHS devoted to improving children and young people's function in school, we had presumed that educational outcomes for those with psychiatric diagnosis were salient to 'a medical purpose.' As a result, we underestimated the need to demonstrate to the CAG that educational performance (attainment, attendances and exclusions) were viewed by researchers, and NHS clinicians working within CAMHS, as key medical outcomes. Also, we had not made a clear enough case for using the linked educational data to examine the aetiological factors for child onset psychiatric disorders. These issues were addressed in the revised scientific proposal, largely by describing research that would examine the bi-directional associations between educational performance and mental health disorders.

In terms of gathering evidence for support of the public benefit to use patient identifiable data via CRIS to link to the national pupil database without patient or caregiver consent, we consulted several clinical, patient and caregiver groups. We gave presentations and collected minutes from the SLaM child and adolescent psychiatry executive group, the Service User Research Enterprise group (SURE), the service user led CRIS Oversight Committee, and SLaM-involved parents, through the BRC patient engagement programme.[5] Because of the focus of one of the projects using the linked data was an investigation into the educational outcomes of children and young people with Autism Spectrum Disorders, we also invited comments on the proposal from the National Autistic Society.

Identifying a trusted third party for managing health data linkages

To address the second issue, we provided an overview to the CAG of the advantages and disadvantages of using NHS Digital as a trusted third party to conduct linkages between SLaM and NPD data. We acknowledged that using NHS Digital would not require SLaM to release patient identifiers of over 35,500 names and addresses to the DfE. However, we described this advantage as fairly limited. We argued that the method proposed would involve no release of clinical data to the DfE, and that mental health status data were already collected and available to informaticians working in DfE National Pupil Database Team under their Special Education Need fields. In addition, we explained that DfE informaticians were already contracted to work with highly sensitive information at an individual level (for example, child protection status, benefit status of parents etc.) under comparable data governance standards expected of NHS Digital informaticians, as detailed by HMG Security Policy Framework v10 2013 (SPF).[6] We acknowledged that an additional potential benefit to using NHS Digital was that patient identifiers would be retained within a NHS environment. But after we invited Department of Health (DoH) and DfE to discuss Information Governance standards between their respective departments (in this case HSCIC and DfE Data Division) they advised, and the data controllers accepted, that there was little difference in data security policy. The DoH official responsible for NHS Digital Information Security and Risk Management Policy liaised with the DfE Departmental Security Unit Information Assurance Policy & Governance Team Leader, and reviewed the DfE Data and Statistics Division internal data processing, information handling controls, and assurance regimes. DoH confirmed that the DfE were in line with government standards and meet equivalent to IG expectations for NHS care system organisations.[7]

To provide further argument for not using NHS Digital as the trusted third party in this linkage, we described two alternatives routes, where NHS Digital performed the linkage and avoided transfer of NHS identifiers to the DfE. One route involved NHS Digital receiving all 15 million identifiers from the DfE, conducting the complex matching with the SLaM identifiers, completing the anonymisation process, and then providing a pseudo-anonymised dataset to SLaM. The second route involved NHS Digital receiving 15 million identifiers from the DfE, conducting the matching process, sending SLaM the controls and cases table with matched SLaM & NPD pseudonyms, and then sending controls and cases with just NPD pseudonym (the DfE remain blinded to SLaM case status) back to the DfE. After this, the DfE would then have to match the education variables of interest on the NPD pseudonym to create a pseudo-anonymised NPD variables table, and finally, send the pseudo-anonymised NPD variables to the SLaM CDLS for later matching with CRIS data. Both SLaM and DfE data controllers were concerned with the number of identifiers that would need to be transferred in both these processes, with sensitive educational variables being conveyed twice between the parties (DfE to NHS Digital, NHS Digital to SLaM CDLS). In addition, for both options DfE would need to supply identifiers for over 15 million individuals to NHS Digital, which may have contained a number of different addresses for each individual, and then separately convey over 500 education variables per individual, linked by pseudonym to the identifiers. DfE and SLaM data controllers, both expressed concerned that the harm caused to individuals if a breach of data security occurred in either of these processes could be significant, especially given the scale and sensitivity of the educational data, and the very large number of individuals involved. Hence, we advised the HRA CAG that both data controllers preferred to pursue a simpler linkage method, using the DfE to undertake the linkage of identifiers, within their secure environment and with appropriate governance controls using the minimum number of identifiers required.

Equivalence in data security requirements between health and education systems

This third issue was largely addressed by demonstrating data security equivalence between the DfE and DoH standards in processing and storing the data. In the re-submission to the CAG we confirmed that all personal identifiers were destroyed immediately after linkage and validation by the DfE, and that data was to be anonymised and only analysed within the same secure environment. The table linking NPD and CRIS pseudonyms, would be destroyed after 60 days from SLaM CDLS receiving the data, to permit some additional data cleaning and validation checks. With these additional details, the application was re-submitted and approved (ref CAG 9-08(a)/2013 0048).[8]

Completing the Memorandum of Understanding between Data Controllers

It took some time to formalise a Memorandum of Understanding (MoU) between the DfE and SLaM. This was due to it being the first time an NHS trust in England had entered into a data sharing contract with the DfE, and the lawyers representing both parties took time to become familiar with the legal basis for sharing data in the proposed manner. After a year under legal review, a signed agreement was eventually completed. One of the areas of contention regarded cross-indemnity. Standard legal advice for commercial data sharing often stipulate that each party should indemnify, and keep indemnified the other party, against any claims brought against them despite the proper performance of the Data Activities as envisaged by the MoU. So, taking this linkage project as an example, if someone were to legally challenge SLaM for data that related to the DfE, which they held temporarily during the matching process, then SLaM would honour an agreement to respond the challenge, and vice versa with the DfE. However, if responsibility was shared between parties, it could have potentially created problems in terms of interpretation, especially in relation to data protection compliance, especially for tasks that are time sensitive such as responding to subject access requests. We eventually reached an agreement that the parties would self–indemnify. This decision was aided by the data flows which provided a clear demarcation between DfE and SLaM data systems and procedures, which we came to understand was important when undertaking data processes on behalf of the other data controller. As SLaM and DfE responsibilities for the project were well defined, both agreed that if one party failed in its obligations, it was most likely that enforcement action would be carried out against the party that was in breach of their agreed obligations at that point in the linkage process.

REFERENCES

1 Department for Education. National Pupil Database User Guide. UK Government, 2015 https://www.gov.uk/government/uploads/system/uploads/attachment_data/file/261189/NPD_User_Guide.pdf (accessed May 3, 2018).
2 Health Research Authority. Section 251 and the Confidentiality Advisory Group (CAG). Health Res. Auth. http://www.hra.nhs.uk/about-the-hra/our-committees/section-251/ (accessed May 22, 2018).
3 Health Research Authority. Principles of Advice: Exploring the concepts of public interest and reseasonably practicable. .
4 NHS Digital. https://digital.nhs.uk/home (accessed May 20, 2018).
5 King's College London - Service User Research Enterprise (SURE). https://www.kcl.ac.uk/ioppn/depts/hspr/research/ciemh/sure/SURE.aspx (accessed May 22, 2018).
6 HMG Cabinet Office. Security policy framework. 2016. https://www.gov.uk/government/publications/security-policy-framework (accessed April 8, 2018).
7 Department for Health. NHS Information Governance Toolkit. https://www.igt.hscic.gov.uk/ (accessed April 8, 2018).
8 Health Research Authority. CAG Advice and HRA/SofS Approval Decisions. Health Res. Auth. https://www.hra.nhs.uk/planning-and-improving-research/application-summaries/confidentiality-advisory-group-registers/ (accessed May 22, 2018).

Supplementary Figure 1: Number of accepted first referrals for all children and young people (aged 4 -17) seen by SLaM CAMHS services (Sept 2007 – August 2013)

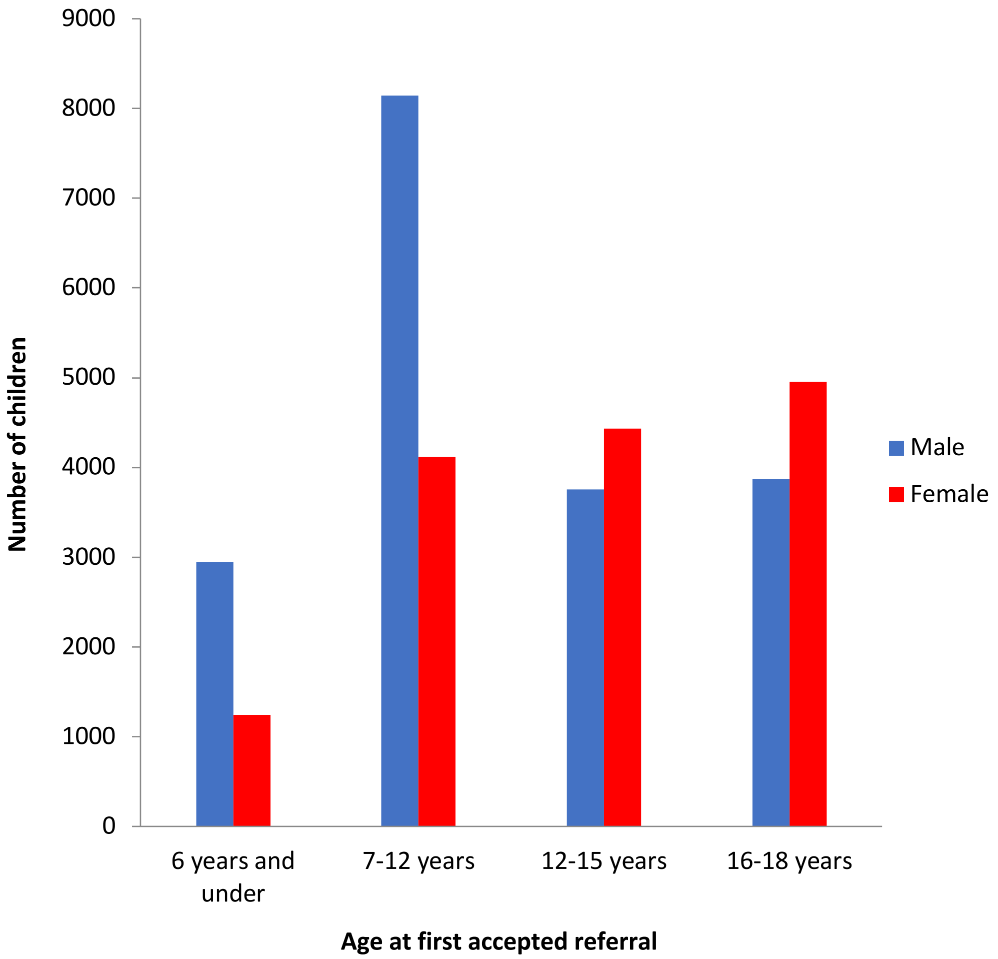

Supplementary Figure 2: Duration between first and last contact with mental health professionals for children and young people (aged 4 -17) accepted to SLAM CAMHS between Sept 2007 – August 2013.

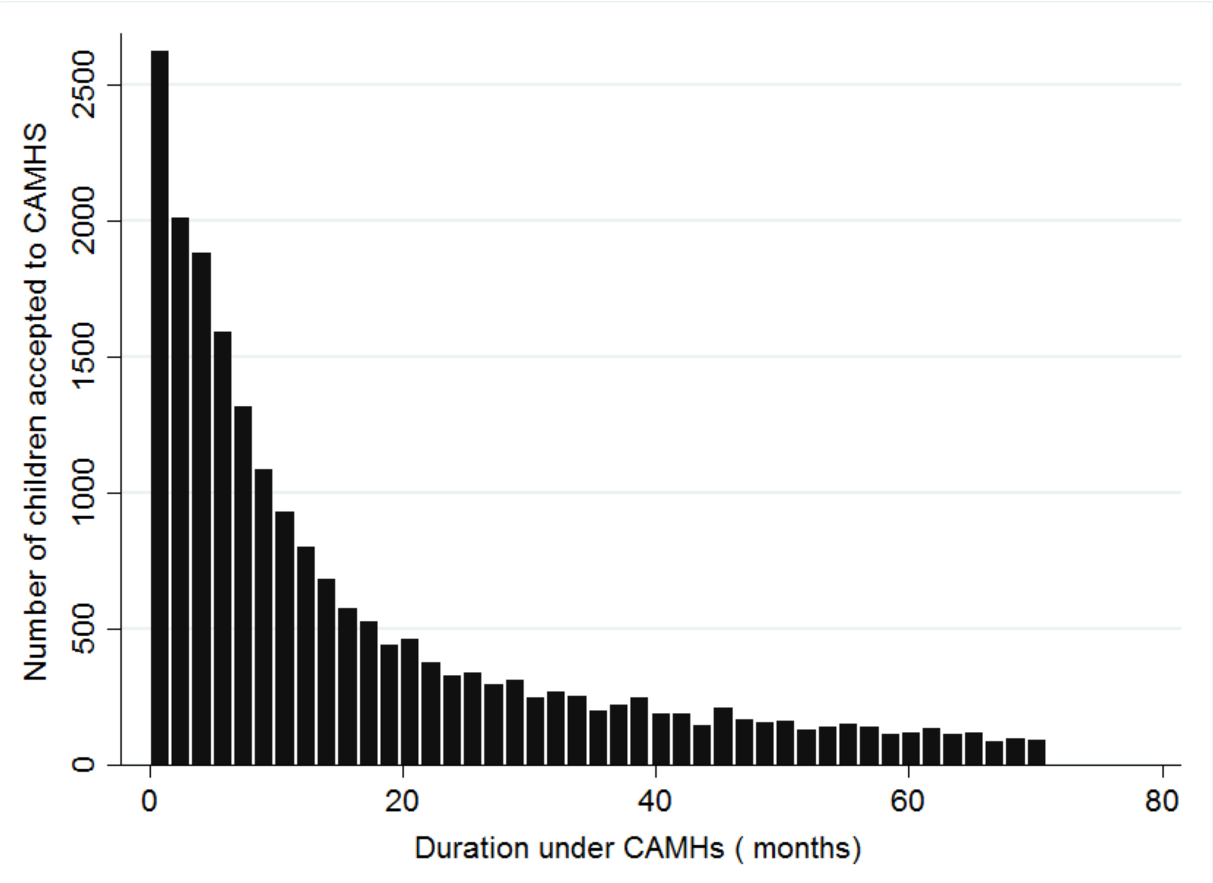

Supplementary table 1: Diagnostic breakdown of all children (aged 4 -17) referred to SLaM CAMHS services between Sept 2007 and August 2013.

ICD-10 Psychiatric Diagnostic Classification		Local Catchment Area*		National Catchment Area*	
		Male (n=15204)	Female (n=11469)	Male (n=4522)	Female (n=4314)
		n (%)	n (%)	n (%)	n (%)
	Any ICD-10 Diagnosis	9315 (61.3)	6587 (57.4)	2592 (57.3)	2545 (59)
Axis One	Pervasive Developmental Disorders (F84)	2116 (13.9)	519 (4.5)	749 (16.5)	248 (5.9)
	Hyperkinetic Disorders (F90)	2345 (15.4)	435 (3.8)	801 (17.7)	210 (4.9)
	Conduct Disorders (F91)	2160 (14.2)	983 (8.6)	392 (8.7)	169 (3.9)
	Disorders due to psychoactive substance use (F10–F19)	253 (1.7)	180 (1.6)	112 (2.5)	53 (1.2)
	Psychotic Disorders (F20-F29, F30-F31, F32.3)	437 (2.9)	438 (3.8)	239 (5.3)	239 (5.5)
	Depression and other (affective) disorders (F32–F39)	733 (4.8)	1497 (13.1)	197 (4.4)	511 (11.8)
	Emotional and stress related disorders (F40-F48, F93, F94, F98)	2442 (16.1)	2930 (25.5)	522 (11.5)	879 (26.4)
	Post-Traumatic Stress Disorder (F43)	269 (1.8)	330 (2.9)	64 (1.4)	105 (2.7)
	Obsessive Compulsive Disorder (F42)	201 (1.3)	220 (1.9)	269(5.9)	164 (3.9)
	No recorded Axis One Diagnosis	5889 (38.7)	4882 (42.6)	1929 (42.7)	1770 (41.0)
Axis Two	Disorders of Scholastic Development (F80-F89)	1048 (6.9)	337 (2.9)	195 (4.3)	89 (3.1)
Axis Three	Intellectual Disorders (F70-F79)	870 (5.7)	357 (3.1)	443 (9.7)	195 (3.8)

*Note: The sample are split by residence, either within 4 London Boroughs served by local SLaM services (Local Catchment area), or from rest of England served by SLaM National and Specialist services (National Catchment Area)
