# Supplementary material for: An approach to linking education, social care and electronic health records for children and young people in South London: a linkage study of child and adolescent mental health service data
Source: BMJ Open. 2019 Jan 29;9(1):e024355. doi: 10.1136/bmjopen-2018-024355 (PMC6352796; doi:10.1136/bmjopen-2018-024355)
Supplement: Author's manuscript [file bmjopen-2018-024355.draft_revisions.pdf]

BMJ Open is committed to open peer review. As part of this commitment we make the peer review history of every article we publish publicly available.

When an article is published we post the peer reviewers' comments and the authors' responses online. We also post the versions of the paper that were used during peer review. These are the versions that the peer review comments apply to.

The versions of the paper that follow are the versions that were submitted during the peer review process. They are not the versions of record or the final published versions. They should not be cited or distributed as the published version of this manuscript.

BMJ Open is an open access journal and the full, final, typeset and author-corrected version of record of the manuscript is available on our site with no access controls, subscription charges or pay-per-view fees (http://bmjopen.bmj.com).

If you have any questions on BMJ Open's open peer review process please email info.bmjopen@bmj.com

BMJ Open

An approach to linking education, social care and electronic health records for children and young people attending mental health services

Journal:	*BMJ Open*
Manuscript ID	bmjopen-2018-024355
Article Type:	Research
Date Submitted by the Author:	25-May-2018

Complete List of Authors:	Downs, Johnny; Kings College London, Institute of Psychiatry, Psychology and Neuroscience<br>Ford, Tamsin; University of Exeter, Exeter Medical School<br>Stewart, Robert; King's College London, Institute of Psychiatry<br>Shetty, Hitesh; South London and Maudsley NHS Foundation Trust, Biomedical Research Centre Nucleus<br>Little, Ryan; Kings College London, Institute of Psychiatry, Psychology and Neuroscience<br>Jewell, Amelia; South London and Maudsley NHS Foundation Trust, BRC Nucleus<br>Broadbent, Matthew; South London and Maudsley NHS Foundation Trust,<br>Deighton, Jessica; University College London, Evidence Based Practice Unit<br>Mostafa, Tarek; Unversity College London, Insitute of Education, Department of Social Science<br>Gilbert, Ruth; UCL Institute of Child Health, Centre for Paediatric Epidemiology and Biostatistics<br>Hotopf, Matthew; King's College London (Institute of Psychiatry),<br>Hayes, Richard; Institute of Psychiatry, Kings College London, Psychological Medicine

Keywords:	Child & adolescent psychiatry < PSYCHIATRY, Health informatics < BIOTECHNOLOGY & BIOINFORMATICS, EPIDEMIOLOGY, Data linkage, School and Education

SCHOLARONE™
Manuscripts

An approach to linking education, social care and electronic health records for children and young people attending mental health services

Johnny M. Downs,[1,2*] Tamsin Ford,[3] Robert Stewart,[1,2] Hitesh Shetty,[2] Ryan Little, [3] Amelia Jewell, [2] Matthew Broadbent, [2] Jessica Deighton,[4] Tarek Mostafa,[5] Ruth Gilbert, [6] Matthew Hotopf[**] and Richard Hayes[1,2**]

* Corresponding author contact information:

Dr Johnny Downs, Department of Child and Adolescent Psychiatry, IOPPN Biomedical Research Centre Nucleus, Ground Floor Mapother House PO BOX 92, King's College London, De Crespigny Park, Denmark Hill, London SE5 8AF. Tel: +44 (0)20 3228 8553. Email: johnny.downs@kcl.ac.uk

**Both authors contributed equally to this work

Author details

[1]Institute of Psychiatry, Psychology Neuroscience, King's College London, UK. [2] NIHR South London and Maudsley NHS Foundation Trust Biomedical Research Centre, London UK. [3]University of Exeter Medical School, UK. [4]Evidence Based Practice Unit, UCL and Anna Freud Centre, London, UK. [5] UCL Institute of Education, University College London, London, UK. [6] Administrative Data Research Centre for England, UCL Great Ormond Street Institute of Child Health, UK.

For peer review only

ABSTRACT

Objectives: Creation of linked mental health, social and education records for research to support evidence based practice for regional mental health services.

Setting: The Clinical Record Interactive Search (CRIS) system was used to extract personal identifiers who accessed psychiatric services between September 2007 and August 2013.

Participants: A clinical cohort of 35,509 children and young people (aged 4-17)

Design: Multiple government and ethical committees approved the link of clinical mental health service data to Department for Education (DfE) data on education and social care services. Under robust governance protocols, fuzzy and deterministic approaches were used by the DfE to match personal identifiers (names, date of birth, and postcode) from NPD and CRIS data sources.

Outcome measures: Risk factors for non-matching to NPD were identified, and the potential impact of non-match biases on ICD-10 mental disorder and persistent school absence (<80% attendance) were examined. Probability weighting and adjustment methods were explored as methods to mitigate the impact of non-match biases.

Results: Governance challenges included developing a research protocol for data linkage which met the legislative requirements for both NHS and DfE. From CRIS 29,278(82.5%) were matched to NPD school attendance records. Presenting to services in late adolescence (aO.R 0.67, 95% C.I 0.59-0.75) or outside of school census timeframes (aO.R 0.15, 0.14-0.17) reduced likelihood of matching. After adjustments for linkage error, ICD-10 mental disorder remained significantly associated with persistent school absence (aO.R 1.13, 1.07-1.22)

Conclusions: The work described sets a precedent for education data being used for medical benefit in England. Linkage between health and education records offers a powerful tool for evaluating the impact of mental health on school function, but biases due to linkage error may produce misleading results. Collaborative research with data providers is needed to develop linkage methods that minimize potential biases in analyses of linked data.

For peer review only

Strengths and limitations of this study’

- This linkage work sets a precedent for education data being used for patient or medical benefit in England.
- It is one of the few studies which examines linkage errors in children and young people, especially where the non-linked group are not subject to consent related bias.
- It provides an example of how potential non-random loss between routinely collected health and non-health linked data can be adjusted by weighting techniques.
- Given the constraints of the data available sharing between data controllers, we were unable to assess false positive matching.
- It was not possible to determine who was not eligible for matching due to complete private or home school educational provision

For peer review only

INTRODUCTION

Large scale longitudinal cohort studies and clinical databases are essential tools for understanding the aetiology and outcomes of childhood mental and physical disorders, including rare or late adverse effects of treatments. However, maintaining the methodological quality of these studies is costly. For example, in the early 1990's the cost of setting up and sustaining the 15,000 families recruited to Avon Longitudinal Study of Parents and Children birth cohort study was around £1 million per year;[1] few existing longitudinal studies are similarly resourced to sustain representation of their target population.[2] Sample attrition during follow up can introduce significant methodological biases and can undermine the validitity of investigations into novel risk-outcome effects.[3] Administrative records from health, education and social public services do not suffer from the same attrition biases by capturing all those receiving a service.[4] They are becoming increasingly available for research : initiatives in Wales and Scotland, have now created linked datasets derived from these data resources, and are using them to help direct local and national public health strategy.[5]

As yet, the potential gains from these 'big data' systems to drive local population-based analyses to improve child public mental health and educational services remain unrealised in England. Linkage of routinely collected data from public services has the potential to improve how local health, education and social care are delivered to children and young people. Certainly, all mental health services, hospital-based child health services, schools and child protection services which serve the same local area could be more efficient if the design, monitoring, targeting and integration of services were based on data.[6] The ethical and legal processes to do this, as well as the technical security requirements, to gain exemption from individual consent for health data are stringent.[7] Even once these challenges have been met, data matching processes can introduce challenges for health service researchers. For example, the introduction of bias by missed matches, particularly if risk factors are both associated with missed matched records and important outcomes, can impact the validity of research findings derived from linked data.[8] This is more likely to occur when linking routinely collected data without a shared identification number (such as health and education records).[9]

In this study we show how an individual NHS trust, with coverage of a geographically defined catchment of 1.2 million, ~190,000 children and young people (South London and Maudsley NHS Foundation Trust, SLaM) developed a sustainable approach to link and anonymise

For peer review only

individual children and young people's records from healthcare, social and educational services. We show how a linkage environment that conformed to NHS and Department for Education (DfE) safeguards was used to build a data resource between a NHS child and adolescent mental health service (CAMHs) records via Clinical Record Interactive Search (CRIS) system[10] linked to the DfE's National Pupil Database (NPD).[11]

This study had two aims: the first was to provide a narrative description of the challenges in gaining approval for a research protocol which needed to meet the legislative requirements for both section 251 of the NHS Act 2006, via recommendation from NHS Health Research Authority Confidentiality Advisory Groups,[7] and The Education (Individual Pupil Information) (Prescribed Persons) (England) Regulations 2009[12] and subsequent amendments[13] - also demonstrating how the legal basis for the 'public benefit' can be made to satisfy General Data Protection Regulations (GDPR).[14] A second aim was to identify the socio-demographic and clinical factors risk factors, within a NHS CAMHS cohort, that were associated with non-matching with DfE educational records. As an applied example , we used the linked data resource to examine how non-matching may have impacted potential associations between child health factors and school absence (i.e. a key education outcome), and how statistical approaches could reduce the effects of this bias.

METHODS

The data resources

NHS Child and Adolescent Mental Health Service Data

SLaM provides comprehensive CAMHS to a geographic catchment of approximately 190,000 children and young people in four south London boroughs— Croydon, Lambeth, Lewisham and Southwark— as well as some specialist services which also accept referrals from outside the four-borough catchment area. Clinical records have been fully electronic across SLaM services since 2007. The process by which CRIS permits these data to be available for research has been described in detail elsewhere.[6,10,15,16] In brief, CRIS extracts information from the electronic health records generated by CAMH services, and by removing personal identifiers, makes pseudo-anonymised data extracts available for analysis by SLaM approved researchers.

CRIS was used to provide an extract of children and young people who were referred to SLaM CAMHS services between 1st September 2007 and August 2013. SLaM has dedicated

For peer review only

multidisciplinary services, which assess and treat school age children and young people under ICD-10 multi-axial classification system. The tables and figures within the supplementary material describe the clinical sample by age and gender first accepted into SLaM CAMHS over a 5-year period. (supplementary table 1 for ICD-10 rates in the clinical sample). As supplementary figure 2-3 shows, the majority of children and young people are first seen in CAMHS services in mid-childhood and will often receive short discreet periods of care. However, some will receive prolonged CAMH services throughout child and adolescence.

Department for Education National Pupil Database

The NPD is a pupil level longitudinal database that matches pupil and school characteristic data to pupil level attainment.(7) The key datasets within the NPD are the pupil census and pupil attainment datasets, which holds data for all assessments that pupils complete during primary and secondary school state education. The census is a snapshot of pupils attending state-maintained schools in England ~ 91% of pupils resident with the SLaM catchment,[17] which is submitted annually on a specific day in January, by a school for all pupils in that school. Pupils held within the NPD are typically aged between 3-19 years, but some from special schools may be up to age 24.

The technical resources

To link CRIS data with other external clinical and non-clinical sources, SLaM developed a research governance model for linking data which satisfies NHS requirements as described in Department of Health Information Governance Review, or 'Caldicott 2' report.[18] In accordance with these guidelines, SLaM set-up the Confidential Data Linkage Service (SLaM CDLS)[10] as a Trusted Third Party or Safe Haven to ensure that confidential patient information can be linked in a way that guarantees the legal and ethical rights of patients and caregivers. A similar provision was available in DfE Data Services Provision which had a linkage service, governed under HMG Security Policy Framework v10 2013 (SPF),[19] with experience of regularly undertaking external linkages with large scale research cohorts including the Millennium Cohort Study[20] and ALSPAC.[1]

Linkage

Preparing the CRIS CAMHS identifiers for matching.

For peer review only

We selected a cohort of young people aged between 4 and 18 years, who were referred to SLaM mental health care between 1st September 2007 and 31st December 2013. As described previously, in the UK, unique identifiers, such as national health identifiers, are not shared between health and education databases, so records require matching on personal identifiers common to both data resources (i.e. names, dates of birth, and residence post code).

Personal identifiers were standardised using the following definitions:

1. **Dob**: format (dd-mm-yyyy)
2. **forename_1**: The first word present in the forename field registered for the individual record. (i.e. all text left of the first white space character in the free text field)
3. **forename_2**: The second word present, if >1 forename present (i.e. second of 2+ names separated by one space or punctuation except "-") (i.e. right of white space)
4. **surname_1**: The first word present in the surname field registered for the individual record. (i.e. all text left of the first white space character)
5. **surname_2**: The second word present, if >1 Surname present (i.e. second word of 2+ names if separated by one space or punctuation except"-")
6. **surname_3**: The whole string in the surname field

Within the longitudinal health record, there were often several different addresses held for each individual. Similarly, there were multiple addresses held for most pupils in the education database. Pupil address data are routinely updated on the 16th January every year. So, we developed a hierarchical system to extract the postcode from the health record most likely to match with education database. Figure 1 shows how this postcode hierarchy might be applied to one individual child, where the blue blocks represent episodes of care provided by CAMHS, and the green time line represents the period of time in school. Taking these considerations into account we produced a hierarchy of postcodes with 1 to 5 levels for each individual seen in CAMHS using logic rules (see figure 1 legend).

A SQL based query was used to extract the identifier data according to these rules. This produced a sample of 36,760 individuals with distinct individual records. Post extraction, we then ran data cleaning and logic checks which included removal of all those with numbers in name string fields (4 cases removed), all those with only one letter in their first or surname (1 case removed), all those with incomplete / atypical English postcodes (214 records hand searched, 77 valid English postcodes were cleaned and retained). We excluded all children whose first referral date was less than 4 years (1095 days) after their Date of birth, unless they had confirmed follow up contact details recorded within the window (i.e. 2007-2013) at least

For peer review only - http://bmjopen.bmj.com/site/about/guidelines.xhtml

one year later than the earliest referral date. This was because clinicians can erroneously record the date of referral or time seen at initial appointment in the date of birth field. This mainly occurs in individuals with only single episodes of contact with services. To fit in with the academic calendar and UK school age, children were then selected if they had their 4th birthday prior to the 1st September 2012. This provided a complete sample of 35,509 ready for matching with the NPD.

All the data prepared for matching had personal identifier fields populated with the exception of the secondary surnames and forenames (i.e. there were no missing values). Dates of Birth ranged from 06/01/1989 – 31/08/2008 which meant that all of these pupils could potentially be found in either current or historic NPD census data. Personal identifiers were standardised to maintain a consistent format with NPD identifiers: SLaM identifiers were prepared to fit with DfE first name, surname and date of birth formats, which included standardising string length, capitalizations, use of spaces, and hyphens.

Only identifiers (names, postcode and date of birth), accompanied by their unique CRIS ID pseudonym, were then sent via secure file transfer to the DfE Data and Statistics Department.

As represented in figure 2 (and described in four stages below) , the DfE matched these against NPD personal identifiers (approximately 15 million records), generating a pupil-specific, non-identifiable NPD ID variable across the whole data set, and adding the CRIS ID to this table for cases only, stripping the resultant table of all identifiers other than the anonymised NPD ID and the pseudonymised CRIS ID, and transferring the data set back to SLaM CDLS using a secure file transfer.

The supplied data items by the SLaM CDLS were matched to the NPD data by DfE informaticians in the stages described below. Initial matching or stage 1 was based on exact matches for the supplied data items. For those cases who did not match at stage 1, stage 2, ‘fuzzy’ matching processes were conducted, and so on, down to stage 4.

- Stage 1: Full match on any combination of CAMHS names (all supplied values including alias), dates of birth and postcode (all supplied) were conducted against the most recent address held, and then the working back, all years/terms of the School census data, Pupil Referral Data, Alternative Provision Data, Early Years Census data. School census data contained preferred and former surnames, which were also searched.

For peer review only

Forenames were checked against forename/middle name combinations.

- Stage 2: Full match on Date of Birth, Postcode and Fuzzy matching on names. To ensure confidence in these matches, results were checked manually. Fuzzy matching was conducted on first two characters of names.

- Stage 3: Full match on names and dates of birth, postcode inward code (the first 2-4 characters) plus first character of the outward code (the latter characters after the space). To ensure confidence in these matches, results were checked manually.

- Stage 4: Full match on names and Postcode with manual check of dates of birth, looking for 'near' dates of birth – where the record may be possibly one year out, one month out, one day out, and transposed month/day.

7.3.4 Analysis of linkage bias

Overall linkage rate was calculated as the percentage of CAMHS individuals linked to any NPD school record on any of the stages 1-4. Potential sources of linkage biases were estimated by comparing linked and unlinked data. For the CAMHS sample described in table 1, we categorised an individual match to NPD school absence data (a subset of the NPD school record) as a binary outcome: match =1, non-match=0. The ICD-10 multi-axial classification system was used to categorise the presence of any recorded mental health diagnosis (i.e. diagnoses status prior to 18th birthday) available between 2007 and 2013.

Using multivariable logistic regression, we explored the associations between a number of risk variables including demographic (e.g. gender, ethnicity, neighbourhood deprivation), clinical (age at first presentation to CAMHS, diagnosis of any ICD-10 disorder) and administrative factors (e.g. postcode hierarchy, see Figure 1) with linkage to the school attendance database as the binary outcome. We used this logistic regression to generate a probability estimate of matching as a function of the risk variables.

Patient and public involvement

In terms of gathering evidence for support of the public benefit to use patient identifiable data via CRIS to link to the national pupil database without patient or caregiver consent, we

For peer review only

consulted several clinical, patient and caregiver groups. We invited comments on privacy notices, gave presentations, and collected minutes from the SLaM child and adolescent psychiatry executive group, the Service User Research Enterprise group (SURE), National Young Persons Advisory Group, the service user led CRIS Oversight Committee, and SLaM-involved parents, through the BRC patient engagement programme.[5] Because of the focus of one of the projects using the linked data was an investigation into the educational outcomes of children and young people with Autism Spectrum Disorders, we also invited comments on the proposal from the National Autistic Society. A lay summary of the purpose of the data linkage was written in collaboration with the Maudsley NIHR service user data linkage advisory group (eg, https://www.maudsleybrc.nihr.ac.uk/facilities/clinical-record-interactive-search-cris/cris-data-linkages/) and a short video was made, to raise awareness of the study and future research plans.

Analysis of linkage error using school attendance outcomes

School absence was chosen as the outcome to assess linkage error because is it challenging to assess the impact of the error for a particular outcome, when there is not an expected one-to-one relationship between one variable and another. For example, when linking patient records to a death registry to determine a patient's survival status, it is difficult to know which matches have been missed – the death registry will only contain patients who have died, and so a non-match could be due to patient being alive or being a missed match.[21] Applying this to school data, there was a need to select a clinically relevant school performance outcome which should be available for all pupils. School attendance should be recorded for all pupils accessing state school, and was clinically relevant, hence useful as outcome for evaluating the impact of linkage error.

For each matched CAMHS-NPD pupil, a binary outcome marker of poor attendance was created for the latest academic year they attended school available between 2007/08 and 2012/13. Pupils were categorised as persistent absentees if they had recorded less than 80% school attendance for the total number of possible school sessions available since their enrolment for that academic year (one session is equal to half a school day).

Using the probability of matching estimate from the linkage bias analysis, we created a weight that was inversely proportional to the probability of being linked to national pupil database

For peer review only - http://bmjopen.bmj.com/site/about/guidelines.xhtml

school attendance data, which was assigned to each individual with linked CAMHS-school absence data. This followed standard methodology for managing non-response bias in conventional cohort and survey designs.[22] Multivariable logistic regression was used to examine predictor variables and association with persistent school absence, initially without weights, and then with inverse probability weights. To examine another approach to adjust for potential selection bias from non-linkage,[23] we examined whether the main effects of interest also persisted after the probability of matching estimate was entered as a covariate in the multivariable logistic regression model.

RESULTS

Section 1: Achieving the ethical, governance and legal approvals

The proposal to link the NPD and CRIS CAMHS data, underwent a robust and lengthy ethical, legal, governance and technical review, conducted by a number of local and national committees within NHS and DfE. Figure 3 provides the timeline and milestones achieved to reach the completion of the linked DfE-SLaM CAMHS dataset. We provide in depth description of the process as a supplementary report to this paper. In brief, gaining the permissions to link the NPD and CRIS CAMHS data was complex, as there was no precedent in England for such a linkage between routinely collected mental health and school data, and there had been no successful completion of linked NHS and non-NHS non health data without individual consent.[24] After a round of discussions between DfE and SLaM, we described a process to link the data, with the main research purpose focused on estimating the effects of clinically recognised, mental health disorder and treatment on educational outcomes. Research Governance approval was granted by the SLaM Caldicott Guardian Committee and the DfE's Data Management Advisory Panel in principle, but the linkage process was contingent on Health Research Authority Confidentiality Advisory Group (HRA CAG) approval.[7]

The HRA CAG rejected the first application, as the research activity proposed did not demonstrate sufficient medical purpose and public benefit to meet the s251 requirements (please see supplementary report for further details). Research conducting a longitudinal analyses of health exposures on education outcomes was not sufficient to meet criteria for conducting research for medical purpose. The HRA CAG also queried whether linkages could not be better carried out using NHS Digital's Trusted Data Linkage Service. The CAG advised that this route would negate the requirement for SLaM to disclose confidential patient

For peer review only - http://bmjopen.bmj.com/site/about/guidelines.xhtml

information to the DfE, and minimise the disclosure of patient information. A final major issue related to the governance arrangements in place around the processing of patient data by the DfE. We hadn't provided sufficient information around retention periods, access arrangements and the extent of identifiable data requested. To prepare for resubmission, we revised the scientific proposal to be more focused on understanding the bi-directional associations between educational performance and mental health disorders. To gather more evidence the public benefit case of scientific proposal, we involved our local NIHR Biomedical Research Centre patient and clinician engagement programme, relevant charitable and education sector bodies.

To address the second issue, we acknowledged that an additional potential benefit to using NHS Digital was that patient identifiers would be retained within a NHS environment, but we were able to confirm that both the SLaM CDLS and DfE were in line with government standards and meet equivalent to information governance (IG) expectations for NHS care system organisations. [25]We also demonstrated, by reviewing the alternative data flows, that using NHS Digital as the trusted third party in this linkage would prove a more complex, and less secure linkage method (please see supplementary report). Briefly, both DfE and SLaM data controllers both expressed concern that the additional step of involving NHS Digital, would significantly increase the potential risk of harm if a breach of data security occurred, especially given the scale and sensitivity of the educational data, and the very large number of individuals involved (over 15 million children).

Section 2: Linkage rates, bias and the impact on education outcome analyses

The overall matching process against any National Pupil Database attendance records provide 29,278 CAMHS-NPD linked records representing a linkage rate of 82.5%. The proportions linked according to DfE matching stages described above: stage 1 - 60.4%; stage 2 - 4.4%; Stage 3 – 1.1% and Stage 4 – 20.7%.

Table 1 identifies the SLaM CAMHS socio-demographic, clinical and administrative record risk factors for linkage to the NPD data. An odds ratio greater than 1 denotes greater chance of successful linkage compared to the reference. In the adjusted model, we found significant differences in most socio-demographic, clinical and administrative factors. Compared to school age children aged under 7, children first referred to CAMHS in late adolescence were significantly less likely to be matched to the NPD, whilst children aged between 7 and 12, were more likely to be successfully matched. Relative to children of White ethnicity, we found other ethnic groups including Asian, Black African and Mixed groups were less likely to be matched. There were no significant differences in successful linkage between children and young people

For peer review only - http://bmjopen.bmj.com/site/about/guidelines.xhtml

in the lowest and highest quartiles of deprivation, but there was significantly reduced linkage success for those living in neighbourhoods in the 2nd and 3rd quartiles. Analyses of the administrative characteristics show that the post codes (which were extracted from clinical episodes of care and did not overlap with January census data (i.e. post codes 2,4 and 5, see figure 1) were less likely to link even after adjustment for other potential explanatory variables (see table 1).

Table 2 provides the socio-demographic, clinical and administrative record characteristics for children and young people seen SLaM CAMHS and the associated risk for persistent absence. The adjusted analyses show that presence of an ICD-10 mental health disorder, age at first referral to CAMHS and Mixed ethnic group (relative to white ethnic groups), were associated with an increased risk of persistent school absence, whilst Asian, Black African, Black Caribbean ethnicity, increased neighbourhood affluence was associated with a decreased risk of persistent absence. These effects persisted after both statistical techniques i) using inverse probability weighting, and ii) adjustment for matching probability were applied to reduce matching bias in the adjusted analyses.

DISCUSSION

We provide the first example of how data linkage projects can be completed using routinely collected NHS and DfE Educational data. This use case demonstrates how the legal basis for the 'public benefit' (i.e. without individual level consent) can be made to satisfy General Data Protection Regulations (GDPR).[14] The regulatory and technical issues for data sharing between health and non-health services are challenging in England, but surmountable. Using deterministic matching techniques provided by the DfE, a large-scale dataset was built between NHS child and mental health data and national school administrative data, providing a linkage for 29,278 patients (82.5% of the NHS cohort) to their educational records. There were significant differences in the socio-demographic and clinical characteristics between matched and non-matched NHS samples. Using these data, we found any child or young person with a ICD-10 mental disorder had approximately 10% greater likelihood of having persistent school absence, when compared to those clinically referred and not meeting threshold for diagnosis. Effects did not change significantly after matching probability adjustment, which suggests these effects on were not driven by selection bias from matching errors.

Analysis of the linkage biases

For peer review only - http://bmjopen.bmj.com/site/about/guidelines.xhtml

Overall, we found 17.5% of the clinical population were not successfully matched to NPD absence data. Whilst enrolment at a non-state maintained school or independent school may explain a proportion,[6,17] a significant minority were likely to match due to administrative factors, which may include missingness or inconsistencies of the matching identifiers, as demonstrated by the effect of post code variation in the analysis, or errors secondary to the matching process. There have been very few studies conducted which examine linkage errors in children and young people, especially where the non-linked group are not subject to consent related bias. Previous research suggests that ethnic minorities are more likely to have administrative records with misspelt names, inaccurately recorded dates of births, and higher levels of residential instability, which may be applicable to this sample.[9,26] These findings provide further argument for greater collaborative research with data providers to develop linkage methods that minimize potential biases in analyses of linked data. [27] Deterministic process which offers little flexibility in matching misspelt names may be a reason why ethnic variation may contribute to missed matches.[9] We found certain age groups, particularly those aged 7 to 12, were associated with a greater likelihood of linkage. This may be due to the greater availability of accurate personal identifiers in the records of this group, as their potential exposure to CAMHS services whilst at school will be longer than other age groups. Similarly, having a ICD-10 mental disorder, which also had an increased likelihood of linking with the school data, may be related to identifier accuracy, as their higher levels of psychopathology will be associated with greater clinical contact, and potentially higher clerical accuracy in recording personal identifiers. It is also more probable that those with higher levels of psychopathology will have longer durations of care that overlap with the school census date.

In our sample, linkage biases appear to have little effect on the association between mental disorder and attendance. However, without information from source data, potential linkage error could be introduced without researchers being aware whether there was need for it to be accounted for in subsequent analyses. Our study highlights the importance of governance arrangements between linkers and analysts to identify which groups are disproportionately affected by linkage error. In our case, this governance has enabled some flexibility with the 'data separation principle' : a common practice in data linkage research, where identifiers (e.g. names or date of birth) are kept separate from attributes (in this case health or education data), to protect privacy and avoid disclosure during the linkage process.[28] While the separation principle might reduce the risk of identification, it does not permit researchers to evaluate the potential risk of linkage bias on future analyses.

For peer review only

Implementation challenges to the data linkage between health and education data

We believe the tasks and challenges to use personal health and education data for data linkage and research can be best described as 'establishing the social license'.[29] This activity included articulating a clear purpose for the linkage, recognized as beneficial by the public or those potentially involved as data subjects, and that the potential risks to individuals or public institutions were tolerable in relation to these benefits. Without the evidence of the proposal being scrutinized and ultimately accepted by those potentially involved as data subjects, and the public institutions/services who act as controllers of the data, it would have been difficult to sustain a case for public benefit – in fact this was one of the reasons why the first application was not approved by the HRA CAG. To prove we had *social licence* to conduct the linkage work, we needed to gather evidence from a number of sources including service users,[33] clinicians, academics, advocacy groups, governance leads; all who may have had a stake in the process and outcomes of the data linkage project.

The second aspect of establishing a social licence, related to fulfilling the professional mandate for properly conducting the linkage process and related research activity. This involved making sure the proposal complied with the known legal, technical and ethical frameworks that governed health data use, and any additional safeguards deemed important by the data controllers and custodians. The technical aspects were not just confined to data security, but also involved preparing the data to ensure the most accurate match, to reduce error and redundancy in later analysis. Fulfilling the mandate also involved the creation of formal contract between the parties involved in controlling, sharing, processing and using the data. This mandate committed us to conduct appropriate analysis and dissemination of the linkage related research, so that we could sustain the social license for future research activity. This may be especially pertinent in England as linkage driven research of routinely collected public service activity is in its infancy, and benefits are yet to be comprehensively established.

Given the time and resources spent to set up this linked data resource, and the potential it holds, it is important that these resources are maintained, and remain accessible for re-use in the future. Without developing specific data sharing agreements between the parties, it can be difficult to establish a collaborative relationship with good governance structures between the controllers, linkers and analysts. Without these structures, there may be a tendency for data controllers to agree to link data only via a 'create and destroy' approach. We believe this maybe unethical in terms of waste and scientifically unsound as prior analyses cannot be re-examined. It also re-exposures data subjects to the potential risks of sharing personal

For peer review only

identifiable information again across different agencies should the linkage need to be repeated in the future.

Strengths and Limitations of the matching methods and matching evaluation

This study has a number of strengths. First it presents a novel application to link data across public sector organisations. The description of the legal, ethical and technical challenges and solutions are described to share some of the lessons we have learned through the process, in the hope that they will be useful for other public organisations. Furthermore, the study provides an example of how potential non-random loss between routinely collected health and non-health linked data can be adjusted by weighting techniques. Because the source data was available data to examine missed linkages, we were able to determine that linkage error did not lead to systematic biases and misleading positive estimates between ICD-10 mental disorder and persistent school absence. The demonstration of matching probability adjustment and inverse probability weighting was intended to illustrate how linkage bias may be reduced, not as a definitive analysis of these data. Future work examining methods to improve linkage techniques, coupled with newer methods for handling uncertainty in analysis of linked data, should help improve the generalisability and quality of future population-based linkage studies.[27] The study also has a number of limitations. We were unable to assess false positive matching, nor able to assess risks for the lower confidence matching (DfE stages 2-4, described above), and the potential effects on school outcome analyses. No shared unique identifier exists between NHS and educational services, nor were their governance arrangements or sufficient resources in place to manually compile a NPD-SLaM CAMHS linked gold-standard data. Another limitation of the matching methodology is the limited number of address identifiers that could be used. For example, due to governance constraints we were unable to use first line of the address, which again limited the capacity to potentially check for coding errors in the postcode. Another contributing factor to linkage error was the age of the child. A substantial number of young people were seen in CAMHS aged 16 and 17 years and would not have data on the NPD if they were no longer attending school. Similarly, we were unable to determine who was not eligible for matching due to complete private or home school educational provision which may be, at greatest, 10% of the sample. All of these limitations are likely to have a led to our finding being an underestimation of the linkage performance.

Implications

For peer review only

The work described sets a precedent for education data being used for patient or medical benefit in England. The regulatory and technical issues for data sharing between health and non-health services are challenging. Certainly, to develop and improve linked data resources, partnerships between academic and government institutions should continue to explore public opinion and develop guidance on building a 'social license' for the sustained use of linked data.[29] In addition, it is important that recent policies which support accessibility for re-use in the future are sustained, especially given the time and resources spent to set up linked data resources, and the potential they hold.[30]

Record linkages are a valuable enhancement to child-based longitudinal studies and clinical registries, which allow evaluation of questions relevant to public health and social care policy. We would urge all mental health trials conducted in children that might influence their attendance or function at school to link to the National Pupil Database. We hope our experience may provide a useful guide for other health services wishing to build information resources using linked administrative data, and specifically to encourage other mental health service providers to work together to link their data to National Pupil Database. In time we hope these resources will generate a wider network of fine-grained data and analytical expertise, which can be used for research to inform commissioning and service provision and better meet children and young person's mental health needs within the population.

Footnotes

- Contributors: The study was conceived by JD, TF and MH. Data extraction was carried out by JD with support from HS, MB, RL and AJ. Data analysis was undertaken by JD. Reporting of findings was led by JD with support from RG,TF, JDe and RH, supervised by RS and MH. All authors contributed to manuscript preparation and approved the final version.
- Role of Funding Source. This work was supported by the Clinical Records Interactive Search (CRIS) system funded and developed by the National Institute for Health Research (NIHR) Mental Health Biomedical Research Centre at South London and Maudsley NHS Foundation Trust and King's College London and a joint infrastructure grant from Guy's and St Thomas' Charity and the Maudsley Charity (grant number BRC-2011-10035). J.D. received supported by a Medical Research Council (MRC) Clinical Research Training Fellowship (MR/L017105/1) and Psychiatry Research Trust Peggy Pollak Research Fellowship in Developmental Psychiatry. RDH was funded by a Medical Research Council (MRC) Population Health Scientist Fellowship (grant number MR/J01219X/1). MH, RS, AS, MB, RL, HS received salary support from the National Institute for Health Research (NIHR) Mental Health Biomedical Research Centre at South London and Maudsley NHS Foundation Trust and King's College London. JDe was supported by the National Institute for Health Research (NIHR) Collaboration for Leadership in Applied Health Research and Care North Thames at

For peer review only - http://bmjopen.bmj.com/site/about/guidelines.xhtml

Bart's Health NHS Trust (NIHR CLAHRC North Thames). RG and JDe are members of the Policy Research Unit in the Health of Children, Young People and Families (CPRU), which is funded by the England Department of Health Policy Research Programme. The views expressed are those of the author(s) and not necessarily those of the NHS, the NIHR or the Department of Health and Social Care.

- Competing Interests : None declared
- Ethics approval: The CRIS data resource received ethical approval as an anonymised data set for secondary analyses from Oxfordshire REC C (Ref: 08/H0606/71+5); and NHS Health Research Authority Confidentiality Advisory Group, reference: CAG 9-08(a)/2014.
- Data sharing statement The data accessed by CRIS remain within an NHS firewall and governance is provided by a patient-led oversight committee. Subject to these conditions, data access is encouraged and those interested should contact RS (robert.stewart@kcl.ac.uk), CRIS academic lead.

For peer review only

REFERENCES

1 Overy C, Reynolds LA, Tansey EM. History of the Avon Longitudinal Study of Parents and Children, C 1980-2000. Queen Mary, University of London, 2012 http://qmro.qmul.ac.uk/xmlui/handle/123456789/2827 (accessed May 22, 2018).

2 Bonevski B, Randell M, Paul C, *et al.* Reaching the hard-to-reach: a systematic review of strategies for improving health and medical research with socially disadvantaged groups. *BMC Med Res Methodol* 2014; **14**: 42.

3 Kho ME, Duffett M, Willison DJ, Cook DJ, Brouwers MC. Written informed consent and selection bias in observational studies using medical records: systematic review. *BMJ* 2009; **338**: b866.

4 Langan SM, Benchimol EI, Guttmann A, *et al.* Setting the RECORD straight: developing a guideline for the REporting of studies Conducted using Observational Routinely collected Data. *Clin Epidemiol* 2013; **5**: 29–31.

5 Public Health Research Data Forum. Enabling Data Linkage to Maximise the Value of Public Health Research Data: full report. Wellcome Trust, 2015 https://wellcome.ac.uk/sites/default/files/enabling-data-linkage-to-maximise-value-of-public-health-research-data-phrdf-mar15.pdf (accessed May 22, 2018).

6 Downs J, Gilbert R, Hayes RD, Hotopf M, Ford T. Linking health and education data to plan and evaluate services for children. *Arch Dis Child* 2017; **102**: 599–602.

7 Health Research Authority. Section 251 and the Confidentiality Advisory Group (CAG). Health Res. Auth. http://www.hra.nhs.uk/about-the-hra/our-committees/section-251/ (accessed May 22, 2018).

8 Lariscy JT. Differential Record Linkage by Hispanic Ethnicity and Age in Linked Mortality Studies Implications for the Epidemiologic Paradox. *J Aging Health* 2011; **23**: 1263–84.

9 Bohensky M. Bias in data linkage studies. In: Harron K, Goldstein H, Dibben C, eds. Methodological Developments in Data Linkage. John Wiley & Sons, Ltd, 2015: 63–82.

10 Perera G, Broadbent M, Callard F, *et al.* Cohort profile of the South London and Maudsley NHS Foundation Trust Biomedical Research Centre (SLaM BRC) Case Register: current status and recent enhancement of an Electronic Mental Health Record-derived data resource. *BMJ Open* 2016; **6**: e008721.

11 Department for Education. National Pupil Database User Guide. UK Government, 2015 https://www.gov.uk/government/uploads/system/uploads/attachment_data/file/261189/NPD_User_Guide.pdf (accessed Jan 3, 2018).

12 The Education (Individual Pupil Information) (Prescribed Persons) (England) Regulations 2009. http://www.legislation.gov.uk/uksi/2009/1563/contents/made (accessed May 22, 2018).

13 The Education (Individual Pupil Information) (Prescribed Persons) (England) (Amendment) Regulations 2013. http://www.legislation.gov.uk/uksi/2013/1193/made (accessed May 22, 2018).

14 Information Commissioner's Office. Guide to the General Data Protection Regulation. 2018 https://ico.org.uk/media/for-organisations/guide-to-the-general-data-protection-regulation-gdpr-1-0.pdf (accessed May 20, 2018).

15 Downs J, Hotopf M, Ford T, *et al.* Clinical predictors of antipsychotic use in children and adolescents with autism spectrum disorders: a historical open cohort study using electronic health records. *Eur Child Adolesc Psychiatry* 2016; **25**: 649–58.

16 Downs JM, Lechler S, Dean H, *et al.* The Association Between Comorbid Autism Spectrum Disorders and Antipsychotic Treatment Failure in Early-Onset Psychosis: A Historical Cohort Study Using Electronic Health Records. *J Clin Psychiatry* 2017; **78**: e1233–41.

For peer review only

17 Department for Education. London Schools and Pupils by Borough and Type of School. 2015 https://data.london.gov.uk/dataset/schools-and-pupils-type-school-borough (accessed May 22, 2018).

18 The Information Governance Review. Information: To share or not to share? Department of Health, UK Government, 2013 https://www.gov.uk/government/uploads/system/uploads/attachment_data/file/192572/2900774_InfoGovernance_accv2.pdf (accessed May 7, 2018).

19 HMG Cabinet Office. Security policy framework. 2016. https://www.gov.uk/government/publications/security-policy-framework (accessed May 8, 2018).

20 Welcome to the Millennium Cohort Study - Centre for Longitudinal Studies. https://www.cls.ioe.ac.uk/page.aspx?sitesectionid=851 (accessed March 26, 2018).

21 Bohensky MA, Jolley D, Sundararajan V, *et al.* Data Linkage: A powerful research tool with potential problems. *BMC Health Serv Res* 2010; **10**: 346.

22 Höfler M, Pfister H, Lieb R, Wittchen H-U. The use of weights to account for non-response and drop-out. *Soc Psychiatry Psychiatr Epidemiol* 2005; **40**: 291–9.

23 Little RJ, Vartivarian S. On weighting the rates in non-response weights. *Stat Med* 2003; **22**: 1589–99.

24 Health Research Authority. CAG Advice and HRA/SofS Approval Decisions. Health Res. Auth. http://www.hra.nhs.uk/about-the-hra/our-committees/section-251/cag-advice-and-approval-decisions/ (accessed May 2, 2018).

25 Department for Health. NHS Information Governance Toolkit. https://www.igt.hscic.gov.uk/ (accessed May 8, 2018).

26 Hagger-Johnson G, Harron K, Goldstein H, Aldridge R, Gilbert R. Probabilistic linkage to enhance deterministic algorithms and reduce data linkage errors in hospital administrative data. *J Innov Health Inform* 2017; **24**: 234–46.

27 Harron K, Goldstein H, Dibben C. Methodological Developments in Data Linkage. Chichester, West Sussex, United Kingdom: Wiley-Blackwell, 2015.

28 Gilbert R, Lafferty R, Hagger-Johnson G, *et al.* GUILD: GUidance for Information about Linking Data sets. *J Public Health Oxf Engl* 2018; **40**: 191–8.

29 Carter P, Laurie GT, Dixon-Woods M. The social licence for research: why care.data ran into trouble. *J Med Ethics* 2015; **41**: 404–9.

30 Administrative Data Research Network. Data Reuse for Research Purposes. Economic and Social Research Council, 2017 https://adrn.ac.uk/media/174422/adrn-034_datareuseforresearchpurposes_02_00_pub.pdf (accessed May 21, 2018).

31 Health Research Authority. Principles of Advice: Exploring the concepts of public interest and reseasonably practicable. http://www.hra.nhs.uk/documents/2014/12/principles-advice-april-2013-v-2.pdf(accessed May 20, 2018).

32 NHS Digital. https://digital.nhs.uk/home (accessed April 26, 2018).

33 King’s College London - Service User Research Enterprise (SURE). https://www.kcl.ac.uk/ioppn/depts/hspr/research/ciemh/sure/SURE.aspx (accessed May 22, 2018).

For peer review only

Table 1: Socio-demographic characteristics of the Child and Adolescent Mental Health sample linked and non-linked to the national pupil database absence data

	Linked pairs (n=29,278)	Non-linked residuals (n=6,231)	O.R (95% C.I.) for +ve linkage	aO.R (95% C.I.)
Male	16,430 (56.1%)	3,296 (52.9)	*Reference*	*Reference*
Female	12,848 (43.9%)	2,935 (47.1)	0.88 (0.83-0.93)**	1.04 (0.97-1.11)
**Age at first referral to mental health services**				
Infant (<7yrs)	3657 (12.5%)	535 (8.7%)	*Reference*	*Reference*
Primary (7-12 yrs)	10,980 (37.5%)	1,284 (20.3%)	1.25 (1.12-1.39)**	1.23 (1.10-1.38)**
Secondary (13-15 yrs)	7,048 (24.1%)	1,140 (18.4%)	0.90 (0.81-1.01)	0.98 (0.88-1.10)
College (16-18)	7570 (25.9%)	3228 (52.2)	0.34 (0.31-0.38)**	0.67 (0.59-0.75)**
**Ethnicity**				
White / White-British	13,838 (47.3%)	2,786 (44.7)	*Reference*	*Reference*
Asian / Asian-British	984 (3.4%)	312 (5.0%)	0.63 (0.56-0.76)**	0.65 (0.56-0.75)**
Black British / African	5,667 (19.4%)	1,181(19.0%)	0.96 (0.89-1.04)	0.82 (0.76-0.89)**
Black British / Afro-Caribbean	1,474 (5.0%)	232 (3.7%)	1.28 (1.11-1.48)**	0.98 (0.84-1.14)
Mixed / Multiple ethnic	2,184 (7.5%)	315 (5.1%)	1.40 (1.23-1.58)**	1.12 (0.99-1.28)
Other ethnic group	1,109 (3.8%)	419 (6.7%)	0.53 (0.47-0.60)**	0.55 (0.48-0.63)**
Not stated	4,022 (13.7%)	986 (15.8%)	0.82 (0.76-0.89)**	0.93 (0.85-1.02)
**Resident within Local catchment area**	22,481 (76.8%)	4,192 (67.2%)	1.61 (1.52-1.71)**	1.04 (0.97-1.12)
**National quartiles of Neighbourhood deprivation**				
1st (Most deprived)	14,398 (49.2%)	2,822 (45.3%)	*Reference*	*Reference*
2nd	9,796 (33.5%)	2,179 (34.9%)	0.88 (0.83-0.94)**	0.90 (0.83-0.96)**
3rd	2,956 (10.1%)	762 (12.2%)	0.76 (0.69-0.83)**	0.81 (0.74-0.89)**
4th (Least Deprived)	2,126 (7.3%)	468 (7.5%)	0.89 (0.79-0.99)*	1.03 (0.92-1.15)
**Address data available2**				
Postcode 1	17,587 (60.1%)	1,987 (31.9%)	*Reference*	*Reference*
Postcode 2	2,956 (10.1%)	990 (15.9%)	0.34 (0.31-0.37)**	0.50 (0.45-0.56)**
Postcode 3	5,776 (19.7%)	1,187 (19.1%)	0.55 (0.51-0.59)**	0.63 (0.58-0.68)**
Postcode 4	1,933 (6.6%)	1,010 (16.2%)	0.22 (0.20-0.23)**	0.35 (0.31-0.39)**
Postcode 5	1,026 (3.5%)	1,057 (17.0%)	0.11 (0.09-0.12)**	0.15 (0.14-0.17)**
**Any ICD-10 Disorder**	17,749 (60.6%)	3,290 (52.8%)	1.38 (1.30-1.45)**	1.11 (1.04-1.18)**

*P < 0.05,** P <0.01
[1]adjusted for all other co-variates listed in the table.
[2] Post code. For a large proportion of cases there are several addresses available for each case. Therefore, postcodes were extracted according to a hierarchy (Postcode 1 being the highest) which we believed to be most likely to have been the place of residence on the day of the 16th Jan 20XX (variable date) census. [See Figure 1 legend]

For peer review only

Table 2: Socio-demographic and odds ratios for persistent (>80%) school absence in 29, 278 children and young people referred to mental health services

	No persistence Absence (n=23,241)	Persistent School Absence (n=5,635)	O.R (95% C.I.)	aO.R[1] (95% C.I.)	Weighted aOR[2]	Match probability adjusted aOR[3]
**Any ICD-10 Disorder**	14,004 (60.2%)	3,594 (63.7%)	1.16 (1.09-1.23)**	1.13 (1.07-1.22)**	1.13 (1.07-1.22)**	1.10 (1.03-1.19)**
**Age at first referral to mental health services**						
<7yrs)	3,031 (13.0%)	298 (5.3%)	Reference	Reference	Reference	Reference
7-12 yrs	9,405 (40.5%)	1,540 (27.3%)	1.67 (1.46-1.90)**	1.67 (1.46-1.90)**	1.67 (1.47-1.91)**	1.60 (1.49-1.84)**
13-15 yrs	5,205 (22.4%)	1,830 (32.5%)	3.58 (3.14-4.07)**	3.65 (3.20-4.18)**	3.71 (3.24-4.23)**	3.66 (3.21-4.18)**
16-18 years	5,600 (24.1%)	1,967 (34.9)	3.57 (3.13-4.06)**	4.20 (3.63-4.86)**	4.15 (3.57-4.81)**	4.70 (3.82-5.78)**
**Female**	10,023 (43.1%)	2,695 (47.8%)	1.20 (1.14-1.28)**	0.97 (0.91-1.03)	0.97 (0.92-1.04)	0.96 (0.91-1.03)
**Ethnicity**						
White / White-British	10,651(45.8%)	3,011(53.4%)	Reference	Reference	Reference	Reference
Asian / Asian-British	815 (3.5%)	159 (2.8%)	0.69 (0.58-0.82)**	0.68 (0.57-0.81)**	0.69 (0.58-0.83)**	0.76 (0.60-0.96)*
Black British / African	4,737 (20.4%)	849 (15.1%)	0.63 (0.58-0.69)**	0.68 (0.62-0.74)**	0.69 (0.63-0.75)**	0.71 (0.64-0.79)**
Black British / Afro-Caribbean	1,213 (5.2%)	248 (4.4%)	0.72 (0.63-0.83)**	0.81 (0.70-0.94)**	0.81 (0.70-0.94)**	0.82 (0.70-0.94)**
Mixed / Multiple ethnic	1,653 (7.1%)	483 (8.6%)	1.03 (0.93-1.15)	1.14 (1.02-1.28)*	1.15 (1.03-1.29)*	1.11 (0.99-1.26)
Other ethnic group	905 (3.9%)	195 (3.5%)	0.76 (0.64-0.89)**	0.78 (0.66-0.92)**	0.80 (0.67-0.96)**	0.92 (0.69-1.22)
Not stated	3,286 (14.1%)	694 (17.4%)	0.74 (0.68-0.82)**	0.78 (0.71-0.86)**	0.79 (0.72-0.87)**	0.79 (0.72-0.87)**
**Resident within Local catchment area**	18,100 (77.8%)	4,064 (72.1%)	0.74 (0.69-0.76)**	0.88 (0.82-0.95)**	0.89 (0.83-0.96)**	0.87 (0.80-0.94)**
**National quartiles of Neighbourhood deprivation**						
1st (Most deprived)	11,326 (79.7%)	2,884 (51.1%)	Reference	Reference	Reference	Reference
2nd	7,891 (33.9%)	1,785 (31.7%)	0.89 (0.83-0.94)**	0.83(0.76-0.89)**	0.82 (0.77-0.88)**	0.85 (0.79-0.92)**
3rd	2,349 (10.1%)	557 (9.9%)	0.93 (0.84-1.03)	0.74(0.69-0.83)**	0.74 (0.66-0.83)**	0.78 (0.69-0.89)**
4th (Least Deprived)	1,692(7.3%)	413 (7.3%)	0.96 (0.85-1.07)	0.70(0.62-0.80)**	0.70 (0.62-0.80)**	0.69 (0.62-0.78)**
**Address data available[4]**						
Postcode 1	14,119(60.7%)	3,170 (56.2%)	Reference	Reference	Reference	Reference
Postcode 2	2,287 (9.8%)	669 (11.9%)	1.30 (1.18-1.43)**	0.71(0.63-0.78)**	0.71 (0.64-0.81)**	0.85 (0.65-1.11)
Postcode 3	4,618 (19.9%)	1,077 (19.1%)	1.03 (0.96-1.12)**	0.92(0.84-0.99)*	0.92 (0.85-1.00)	1.01 (0.87-1.19)
Postcode 4	1,448 (6.2%)	485 (8.6%)	1.49 (1.33-1.67)**	0.81(0.71-0.93)**	0.82 (0.72-0.95)**	1.14 (0.71-1.81)
Postcode 5	788 (3.4%)	238 (4.2%)	1.34 (1.16-1.56)**	0.93(0.79-1.10)	0.93 (0.78-1.09)	1.85 (0.74-4.66)

*P<0.05,**P<0.01, [1]adjusted for all other co-variates listed in the table. [2] adjusted model with inverse probability weighting for matching included, [3]adjusted model with addition of matching probability estimates entered as a co-variate, [4] See Figure 1 legend

For peer review only

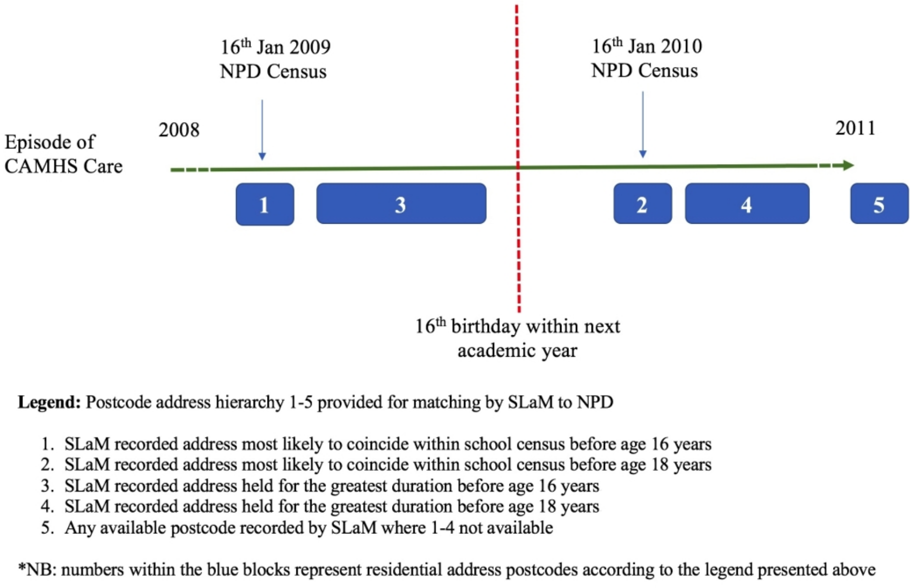

Figure 1: Creating a hierarchy of matching postcodes* to improve the link between CRIS CAMHS Data to DfE National Pupil Database

759x472mm (72 x 72 DPI)

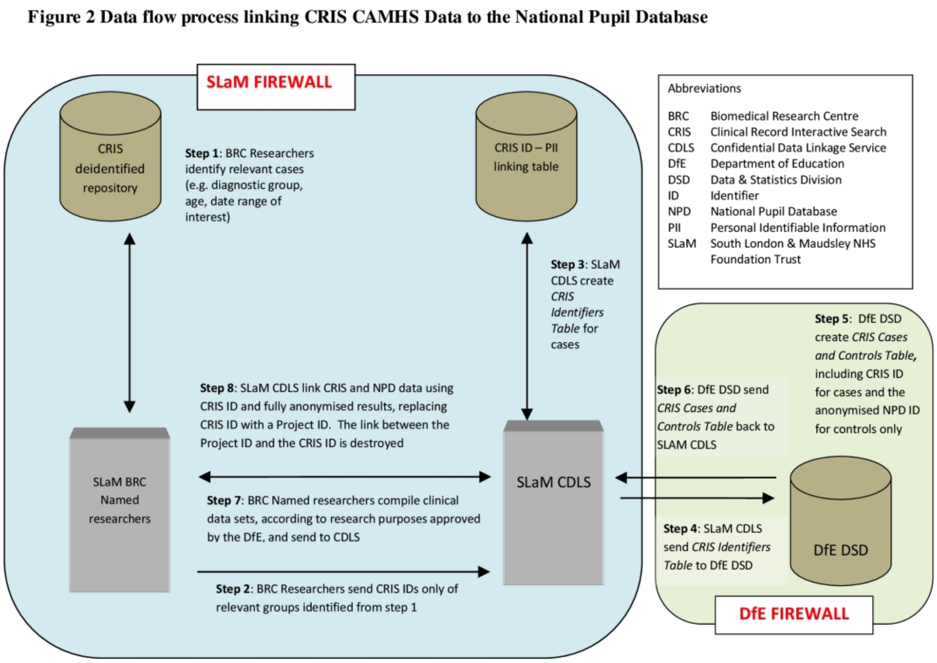

Figure 2: Data flow process linking CRIS CAMHS Data to the National Pupil Database

825x583mm (72 x 72 DPI)

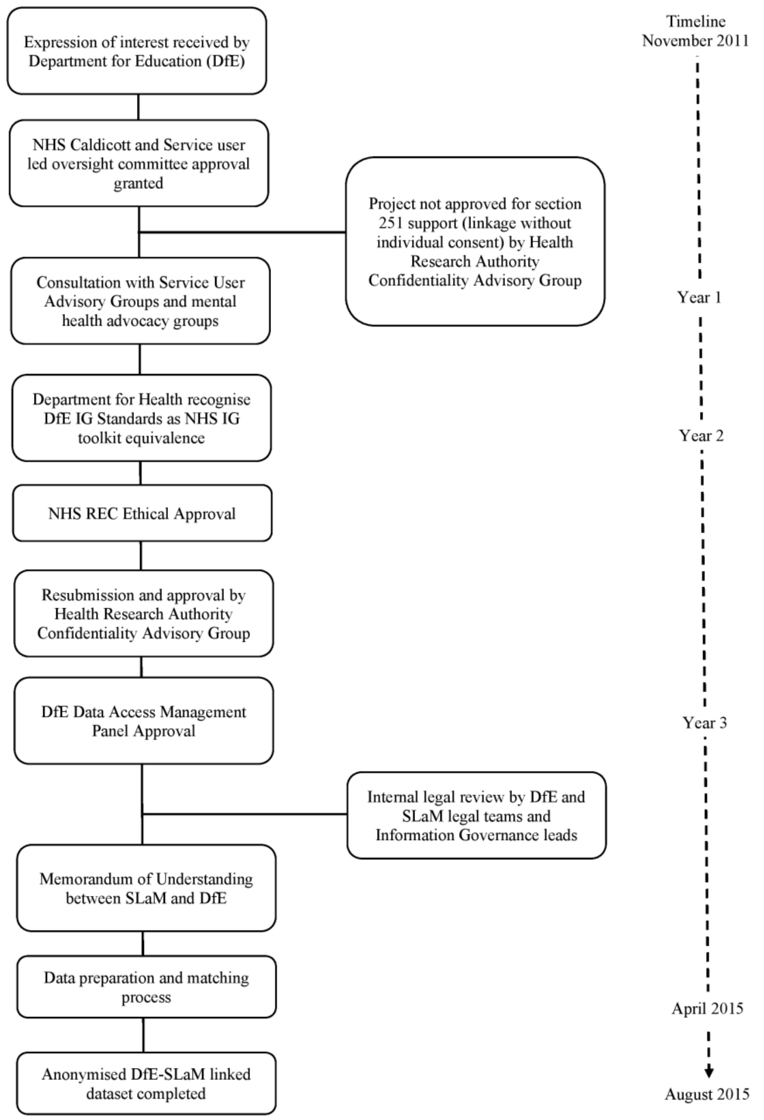

Figure 3: A timeline of the ethical, legal and technical milestones for reaching a data linkage between DfE and SLaM

210x280mm (300 x 300 DPI)

SUPPLEMENTARY MATERIAL

Title: An approach to linking education, social care and electronic health records for children and young people attending mental health services

Johnny Downs, Tamsin Ford, Robert Stewart, Hitesh Shetty, Ryan Little, Amelia Jewell, Matthew Broadbent, Jessica Deighton, Tarek Mostafa, Ruth Gilbert, Matthew Hotopf and Richard Hayes

* Corresponding author contact information:

Dr Johnny Downs, Department of Child and Adolescent Psychiatry, IOPPN Biomedical Research Centre Nucleus, Ground Floor Mapother House PO BOX 92, King's College London, De Crespigny Park, Denmark Hill, London SE5 8AF. Tel: +44 (0)20 3228 8553. Email: johnny.downs@kcl.ac.uk

For peer review only

Supplementary report on providing a linked health and educational data resource: achieving the ethical, governance and legal approvals

Initiating the discussion on the purpose and process of the linkage between public sector data controllers

We first approached the Department for Education directly who held nationally collected education data via termly school submissions to the National Pupil Database.[1] We planned a linkage with national data, as opposed to regional data sources held by the local education authorities, to prevent clinical sample attrition. We expected a considerable proportion of children and young people receiving SLaM treatment would reside outside the SLaM Catchment area or potentially move outside the catchment after treatment. In addition, the Department for Education had relatively transparent systems, and a dedicated office, for managing requests for educational data extracts, through their National Pupil Database Team. Once Research Governance approval was granted by the SLaM Caldicott Guardian Committee and the DfE's Data Management Advisory Panel, we prepared an application to the Health Research Authority Confidentiality Advisory Group (HRA CAG).[2] The HRA CAG have the authority to provide recommendations on behalf of the Secretary of State for Health to permit the linkage of NHS data without individual patient consent for the purposes of research, if it meets the criteria within section 251 of the NHS Act 2006. The main purpose of our application was to examine and

estimate the effects of clinically recognised, mental health disorder and treatment on educational outcomes.

The HRA CAG rejected the first application, as the research activity proposed did not demonstrate sufficient medical purpose and public benefit to meet the s251 requirements. It was highlighted by the HRA CAG that support under current regulations could only be provided where potential public benefit were sufficiently defined.[3] In particular, it was noted that in order to satisfy one of the conditions in schedule 3 of the Data Protection Act (32) (required to process sensitive personal data including data relating to an individual's physical or mental health) a medical purpose would also need to be specified; education outcomes in themselves would not suffice as a medical outcome. A second issue, was the lack of consideration of a practicable alternative to the use of confidential patient information without consent.

The HRA CAG also queried whether we had considered if the Health and Social Care Information Centre (HSCIC, now NHS Digital)[4] could carry out the linkages on the applicant's behalf using their Trusted Data Linkage Service. The CAG advised that this route would negate the requirement for SLaM to disclose confidential patient information to the DfE, and minimise the disclosure of patient information. A final major issue related to the governance arrangements in place around the processing of patient data by the DfE. We hadn't provided sufficient information around retention periods, access arrangements and the extent of identifiable data requested.

Defining 'medical purpose' and public benefit when seeking s251 support

To prepare for resubmission, we examined the issues identified by the HRA CAG. The initial application took a broad interpretation of 'medical purpose.' Given our clinical experience working in CAMHS, and the time CAMHS devoted to improving children and young people's function in school, we had presumed that educational outcomes for those with psychiatric diagnosis were salient to 'a medical purpose.' As a result, we underestimated the need to demonstrate to the CAG that educational performance (attainment, attendances and exclusions) were viewed by researchers, and NHS clinicians working within CAMHS, as key medical outcomes. Also, we had not made a clear enough case for using the linked educational data to examine the aetiological factors for child onset psychiatric disorders. These issues were addressed in the revised scientific proposal, largely by describing research that would examine the bi-directional associations between educational performance and mental health disorders.

For peer review only

In terms of gathering evidence for support of the public benefit to use patient identifiable data via CRIS to link to the national pupil database without patient or caregiver consent, we consulted several clinical, patient and caregiver groups. We gave presentations and collected minutes from the SLaM child and adolescent psychiatry executive group, the Service User Research Enterprise group (SURE), the service user led CRIS Oversight Committee, and SLaM-involved parents, through the BRC patient engagement programme.[5] Because of the focus of one of the projects using the linked data was an investigation into the educational outcomes of children and young people with Autism Spectrum Disorders, we also invited comments on the proposal from the National Autistic Society.

Identifying a trusted third party for managing health data linkages

To address the second issue, we provided an overview to the CAG of the advantages and disadvantages of using NHS Digital as a trusted third party to conduct linkages between SLaM and NPD data. We acknowledged that using NHS Digital would not require SLaM to release patient identifiers of over 35,500 names and addresses to the DfE. However, we described this advantage as fairly limited. We argued that the method proposed would involve no release of clinical data to the DfE, and that mental health status data were already collected and available to informaticians working in DfE National Pupil Database Team under their Special Education Need fields. In addition, we explained that DfE informaticians were already contracted to work with highly sensitive information at an individual level (for example, child protection status, benefit status of parents etc.) under comparable data governance standards expected of NHS Digital informaticians, as detailed by HMG Security Policy Framework v10 2013 (SPF).[6] We acknowledged that an additional potential benefit to using NHS Digital was that patient identifiers would be retained within a NHS environment. But after we invited Department of Health (DoH) and DfE to discuss Information Governance standards between their respective departments (in this case HSCIC and DfE Data Division) they advised, and the data controllers accepted, that there was little difference in data security policy. The DoH official responsible for NHS Digital Information Security and Risk Management Policy liaised with the DfE Departmental Security Unit Information Assurance Policy & Governance Team Leader, and reviewed the DfE Data and Statistics Division internal data processing, information handling controls, and assurance regimes. DoH confirmed that the DfE were in line with government standards and meet equivalent to IG expectations for NHS care system organisations.[7]

To provide further argument for not using NHS Digital as the trusted third party in this linkage, we described two alternatives routes, where NHS Digital performed the linkage and avoided transfer of NHS identifiers to the DfE. One route involved NHS Digital receiving all 15 million identifiers from the DfE, conducting the complex matching with the SLaM identifiers, completing the anonymisation process, and then providing a pseudo-anonymised dataset to SLaM. The second route involved NHS Digital receiving 15 million identifiers from the DfE, conducting the matching process, sending SLaM the controls and cases table with matched SLaM & NPD pseudonyms, and then sending controls and cases with just NPD pseudonym (the DfE remain blinded to SLaM case status) back to the DfE. After this, the DfE would then have to match the education variables of interest on the NPD pseudonym to create a pseudo-anonymised NPD variables table, and finally, send the pseudo-anonymised NPD variables to the SLaM CDLS for later matching with CRIS data. Both SLaM and DfE data controllers were concerned with the number of identifiers that would need to be transferred in both these processes, with sensitive educational variables being conveyed twice between the parties (DfE to NHS Digital, NHS Digital to SLaM CDLS). In addition, for both options DfE would need to supply identifiers for over 15 million individuals to NHS Digital, which may have contained a number of different addresses for each individual, and then separately convey over 500 education variables per individual, linked by pseudonym to the identifiers. DfE and SLaM data controllers, both expressed concerned that the harm caused to individuals if a breach of data security occurred in either of these processes could be significant, especially given the scale and sensitivity of the educational data, and the very large number of individuals involved. Hence, we advised the HRA CAG that both data controllers preferred to pursue a simpler linkage method, using the DfE to undertake the linkage of identifiers, within their secure environment and with appropriate governance controls using the minimum number of identifiers required.

Equivalence in data security requirements between health and education systems

This third issue was largely addressed by demonstrating data security equivalence between the DfE and DoH standards in processing and storing the data. In the re-submission to the CAG we confirmed that all personal identifiers were destroyed immediately after linkage and validation by the DfE, and that data was to be anonymised and only analysed within the same secure environment. The table linking NPD and CRIS pseudonyms, would be destroyed after 60 days from SLaM CDLS receiving the data, to permit some additional data cleaning and validation checks. With these additional details, the application was re-submitted and approved (ref CAG 9-08(a)/2013 0048).[8]

For peer review only - http://bmjopen.bmj.com/site/about/guidelines.xhtml

Completing the Memorandum of Understanding between Data Controllers

It took some time to formalise a Memorandum of Understanding (MoU) between the DfE and SLaM. This was due to it being the first time an NHS trust in England had entered into a data sharing contract with the DfE, and the lawyers representing both parties took time to become familiar with the legal basis for sharing data in the proposed manner. After a year under legal review, a signed agreement was eventually completed. One of the areas of contention regarded cross-indemnity. Standard legal advice for commercial data sharing often stipulate that each party should indemnify, and keep indemnified the other party, against any claims brought against them despite the proper performance of the Data Activities as envisaged by the MoU. So, taking this linkage project as an example, if someone were to legally challenge SLaM for data that related to the DfE, which they held temporarily during the matching process, then SLaM would honour an agreement to respond the challenge, and vice versa with the DfE. However, if responsibility was shared between parties, it could have potentially created problems in terms of interpretation, especially in relation to data protection compliance, especially for tasks that are time sensitive such as responding to subject access requests. We eventually reached an agreement that the parties would self–indemnify. This decision was aided by the data flows which provided a clear demarcation between DfE and SLaM data systems and procedures, which we came to understand was important when undertaking data processes on behalf of the other data controller. As SLaM and DfE responsibilities for the project were well defined, both agreed that if one party failed in its obligations, it was most likely that enforcement action would be carried out against the party that was in breach of their agreed obligations at that point in the linkage process.

For peer review only

REFERENCES

1 Department for Education. National Pupil Database User Guide. UK Government, 2015 https://www.gov.uk/government/uploads/system/uploads/attachment_data/file/261189/NPD_User_Guide.pdf (accessed May 3, 2018).
2 Health Research Authority. Section 251 and the Confidentiality Advisory Group (CAG). Health Res. Auth. http://www.hra.nhs.uk/about-the-hra/our-committees/section-251/ (accessed May 22, 2018).
3 Health Research Authority. Principles of Advice: Exploring the concepts of public interest and reseasonably practicable. .
4 NHS Digital. https://digital.nhs.uk/home (accessed May 20, 2018).
5 King's College London - Service User Research Enterprise (SURE). https://www.kcl.ac.uk/ioppn/depts/hspr/research/ciemh/sure/SURE.aspx (accessed May 22, 2018).
6 HMG Cabinet Office. Security policy framework. 2016. https://www.gov.uk/government/publications/security-policy-framework (accessed April 8, 2018).
7 Department for Health. NHS Information Governance Toolkit. https://www.igt.hscic.gov.uk/ (accessed April 8, 2018).
8 Health Research Authority. CAG Advice and HRA/SofS Approval Decisions. Health Res. Auth. https://www.hra.nhs.uk/planning-and-improving-research/application-summaries/confidentiality-advisory-group-registers/ (accessed May 22, 2018).

For peer review only

Supplementary Figure 1: Number of accepted first referrals for all children and young people (aged 4 -17) seen by SLaM CAMHS services (Sept 2007 – August 2013)

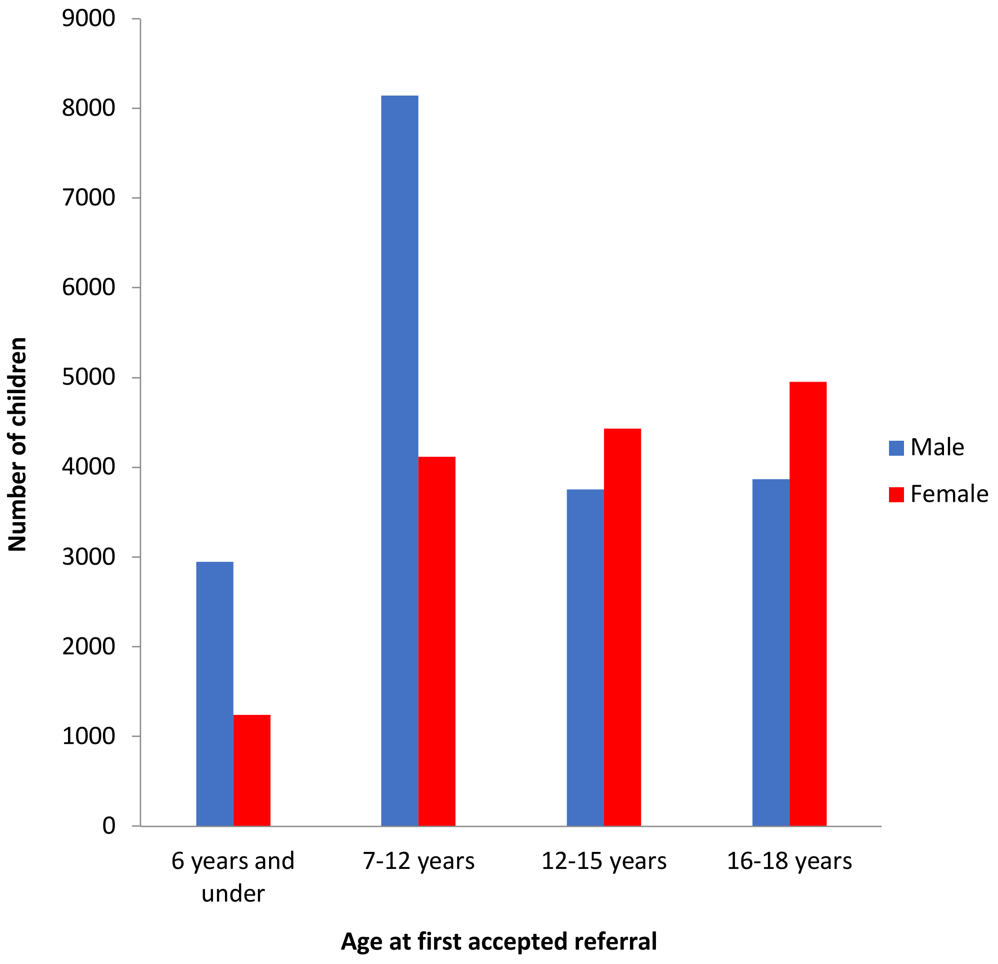

Supplementary Figure 2: Duration between first and last contact with mental health professionals for children and young people (aged 4 -17) accepted to SLAM CAMHS between Sept 2007 – August 2013.

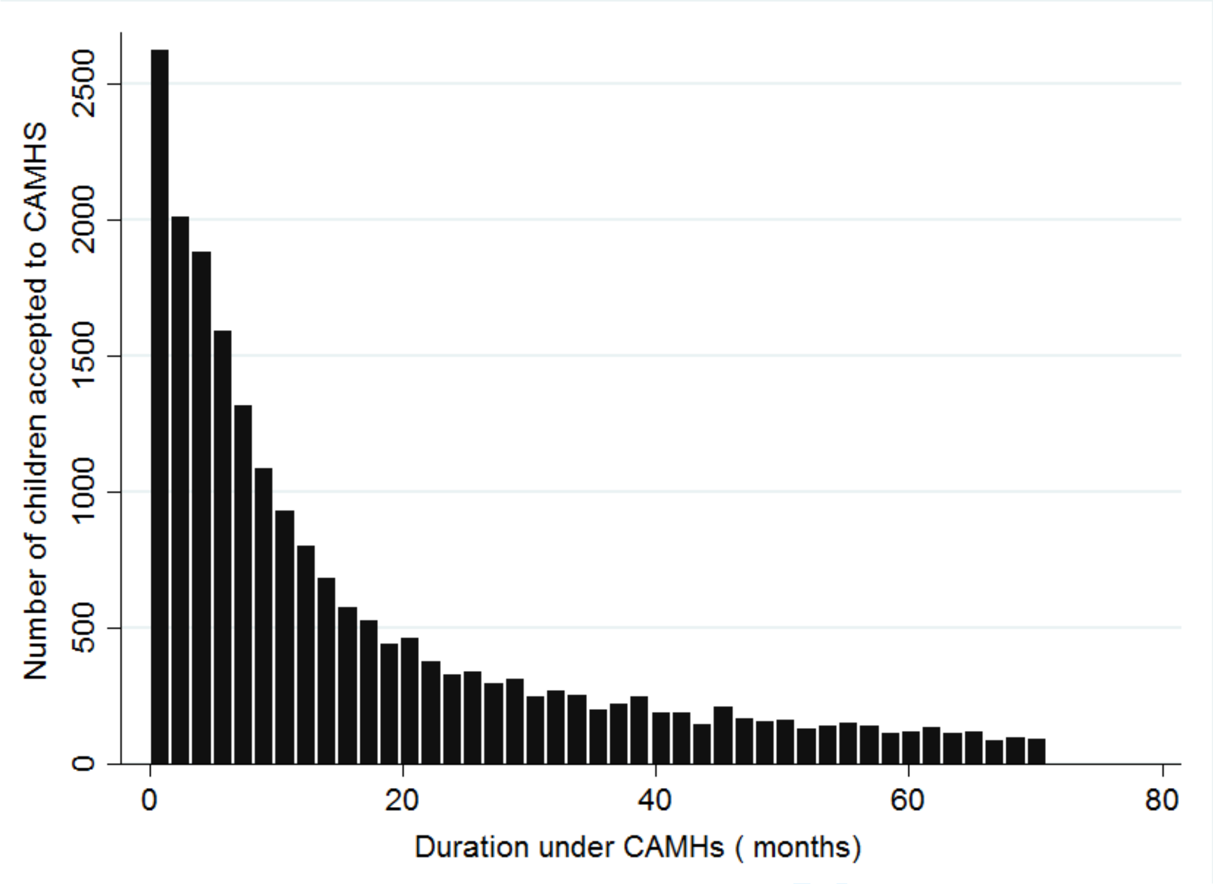

only

Supplementary table 1: Diagnostic breakdown of all children (aged 4 -17) referred to SLaM CAMHS services between Sept 2007 and August 2013.

ICD-10 Psychiatric Diagnostic Classification		Local Catchment Area*		National Catchment Area*	
		Male (n=15204)	Female (n=11469)	Male (n=4522)	Female (n=4314)
		n (%)	n (%)	n (%)	n (%)
	Any ICD-10 Diagnosis	9315 (61.3)	6587 (57.4)	2592 (57.3)	2545 (59)
Axis One	Pervasive Developmental Disorders (F84)	2116 (13.9)	519 (4.5)	749 (16.5)	248 (5.9)
	Hyperkinetic Disorders (F90)	2345 (15.4)	435 (3.8)	801 (17.7)	210 (4.9)
	Conduct Disorders (F91)	2160 (14.2)	983 (8.6)	392 (8.7)	169 (3.9)
	Disorders due to psychoactive substance use (F10–F19)	253 (1.7)	180 (1.6)	112 (2.5)	53 (1.2)
	Psychotic Disorders (F20-F29, F30-F31, F32.3)	437 (2.9)	438 (3.8)	239 (5.3)	239 (5.5)
	Depression and other (affective) disorders (F32–F39)	733 (4.8)	1497 (13.1)	197 (4.4)	511 (11.8)
	Emotional and stress related disorders (F40-F48, F93, F94, F98)	2442 (16.1)	2930 (25.5)	522 (11.5)	879 (26.4)
	Post-Traumatic Stress Disorder (F43)	269 (1.8)	330 (2.9)	64 (1.4)	105 (2.7)
	Obsessive Compulsive Disorder (F42)	201 (1.3)	220 (1.9)	269(5.9)	164 (3.9)
	No recorded Axis One Diagnosis	5889 (38.7)	4882 (42.6)	1929 (42.7)	1770 (41.0)
Axis Two	Disorders of Scholastic Development (F80-F89)	1048 (6.9)	337 (2.9)	195 (4.3)	89 (3.1)
Axis Three	Intellectual Disorders (F70-F79)	870 (5.7)	357 (3.1)	443 (9.7)	195 (3.8)

*Note: The sample are split by residence, either within 4 London Boroughs served by local SLaM services (Local Catchment area), or from rest of England served by SLaM National and Specialist services (National Catchment Area)

GUILD Guidance

Items in the RECORD statement relevant to data linkage (6)[1]

**Title and abstract**<br>RECORD 1.3: If linkage between databases was conducted for the study, this should be clearly stated in the title or abstract. Introduction – **Yes stated in the abstract – page 3**
**Methods: Participants**<br>RECORD 6.3: If the study involved linkage of databases, consider use of a flow diagram or other graphical display to demonstrate the data linkage process, including the number of individuals with linked data at each stage. **Yes, provided in the results (page 12 , and data flow provided in figure 2**
**Methods: Statistical Methods**<br>RECORD ITEM 12.2: Authors should provide information on the data cleaning methods used in the study. **Yes, provided within the methods section**<br>RECORD ITEM 12.3: State whether the study included person-level, institutional-level, or other data linkage across two or more databases. Linkage techniques and methods used to evaluate linkage quality should be provided. **Yes, provided within the methods section (pages 6-9)**
**Results: Participants**<br>RECORD ITEM 13.1: Describe in detail the selection of the persons included in the study (i.e., study population selection), including filtering based on data quality, data availability and linkage. The selection of included persons can be described in the text and/or by means of the study flow diagram.<br>**Yes, provided in pages 12, and table 1 and 2.**
**Discussion: Limitations**<br>Discussion RECORD ITEM 19.1: Discuss the implications of using data that were not created or collected to answer the specific research question(s). Include discussion of misclassification bias, unmeasured confounding, missing data, and changing eligibility over time, as they pertain to the study being reported.<br>**Yes, provided within the discussion section (pages 16)**

Reference

1 Gilbert R, Lafferty R, Hagger-Johnson G, *et al.* GUILD: GUidance for Information about Linking Data sets. *J Public Health Oxf Engl* 2018; **40**: 191–8.

For peer review only

An approach to linking education, social care and electronic health records for children and young people in South London: a linkage study of child and adolescent mental health service data.

Journal:	*BMJ Open*
Manuscript ID	bmjopen-2018-024355.R1
Article Type:	Research
Date Submitted by the Author:	02-Oct-2018

Complete List of Authors:	Downs, Johnny; Kings College London, Institute of Psychiatry, Psychology and Neuroscience<br>Ford, Tamsin; University of Exeter, Exeter Medical School<br>Stewart, Robert; King's College London, Institute of Psychiatry<br>Epstein, Sophie; South London and Maudsley NHS Foundation Trust, NIHR Maudsley Biomedical Research Centre<br>Shetty, Hitesh; South London and Maudsley NHS Foundation Trust, Biomedical Research Centre Nucleus<br>Little, Ryan; Kings College London, Institute of Psychiatry, Psychology and Neuroscience<br>Jewell, Amelia; South London and Maudsley NHS Foundation Trust, BRC Nucleus<br>Broadbent, Matthew; South London and Maudsley NHS Foundation Trust,<br>Deighton, Jessica; University College London, Evidence Based Practice Unit<br>Mostafa, Tarek; Unversity College London, Insitute of Education, Department of Social Science<br>Gilbert, Ruth; UCL Institute of Child Health, Centre for Paediatric Epidemiology and Biostatistics<br>Hotopf, Matthew; King's College London (Institute of Psychiatry),<br>Hayes, Richard; Institute of Psychiatry, Kings College London, Psychological Medicine

**Primary Subject Heading**:	Mental health
Secondary Subject Heading:	Epidemiology, Health informatics, Paediatrics
Keywords:	Child & adolescent psychiatry < PSYCHIATRY, Health informatics < BIOTECHNOLOGY & BIOINFORMATICS, EPIDEMIOLOGY, Data linkage, School and Education

SCHOLARONE™ Manuscripts

An approach to linking education, social care and electronic health records for children and young people in South London: a linkage study of child and adolescent mental health service data.

Johnny M. Downs,[1,2*] Tamsin Ford,[3] Robert Stewart,[1,2] , Sophie Epstein, [1,2] Hitesh Shetty,[2] Ryan Little, [3] Amelia Jewell, [2] Matthew Broadbent, [2] Jessica Deighton,[4] Tarek Mostafa,[5] Ruth Gilbert, [6] Matthew Hotopf[**] and Richard Hayes[1,2**]

* Corresponding author contact information:

Dr Johnny Downs, Department of Child and Adolescent Psychiatry, IOPPN Biomedical Research Centre Nucleus, Ground Floor Mapother House PO BOX 92, King's College London, De Crespigny Park, Denmark Hill, London SE5 8AF. Tel: +44 (0)20 3228 8553. Email: johnny.downs@kcl.ac.uk

**Both authors contributed equally to this work

Author details

[1]Institute of Psychiatry, Psychology Neuroscience, King's College London, UK. [2] NIHR South London and Maudsley NHS Foundation Trust Biomedical Research Centre, London UK. [3]University of Exeter Medical School, UK. [4]Evidence Based Practice Unit, UCL and Anna Freud Centre, London, UK. [5] UCL Institute of Education, University College London, London, UK. [6] Administrative Data Research Centre for England, UCL Great Ormond Street Institute of Child Health, UK.

For peer review only

ABSTRACT

Objectives: Creation of linked mental health, social and education records for research to support evidence based practice for regional mental health services.

Setting: The Clinical Record Interactive Search (CRIS) system was used to extract personal identifiers who accessed psychiatric services between September 2007 and August 2013.

Participants: A clinical cohort of 35,509 children and young people (aged 4-17)

Design: Multiple government and ethical committees approved the link of clinical mental health service data to Department for Education (DfE) data on education and social care services. Under robust governance protocols, fuzzy and deterministic approaches were used by the DfE to match personal identifiers (names, date of birth, and postcode) from NPD and CRIS data sources.

Outcome measures: Risk factors for non-matching to NPD were identified, and the potential impact of non-match biases on ICD-10 mental disorder and persistent school absence (<80% attendance) were examined. Probability weighting and adjustment methods were explored as methods to mitigate the impact of non-match biases.

Results: Governance challenges included developing a research protocol for data linkage which met the legislative requirements for both NHS and DfE. From CRIS 29,278(82.5%) were matched to NPD school attendance records. Presenting to services in late adolescence (aO.R 0.67, 95% C.I 0.59-0.75) or outside of school census timeframes (aO.R 0.15, 0.14-0.17) reduced likelihood of matching. After adjustments for linkage error, ICD-10 mental disorder remained significantly associated with persistent school absence (aO.R 1.13, 1.07-1.22)

Conclusions: The work described sets a precedent for education data being used for medical benefit in England. Linkage between health and education records offers a powerful tool for evaluating the impact of mental health on school function, but biases due to linkage error may produce misleading results. Collaborative research with data providers is needed to develop linkage methods that minimize potential biases in analyses of linked data.

Strengths and limitations of this study'

- This linkage work sets a precedent for education data being used for patient or medical benefit in England.
- It is one of the few studies which examines linkage errors in children and young people, especially where the non-linked group are not subject to consent related bias.
- It provides an example of how potential non-random loss between routinely collected health and non-health linked data can be adjusted by weighting techniques.
- Given the constraints of the data available sharing between data controllers, we were unable to assess false positive matching.
- It was not possible to determine who was not eligible for matching due to complete private or home school educational provision

For peer review only

INTRODUCTION

Large scale longitudinal cohort studies and clinical databases are essential tools for understanding the aetiology and outcomes of childhood mental and physical disorders, including rare or late adverse effects of treatments. However, maintaining the methodological quality of these studies is costly. For example, in the early 1990's the cost of setting up and sustaining the 15,000 families recruited to Avon Longitudinal Study of Parents and Children birth cohort study was around £1 million per year;[1] few existing longitudinal studies are similarly resourced to sustain representation of their target population.[2] Sample attrition during follow up can introduce significant methodological biases and can undermine the validitity of investigations into novel risk-outcome effects.[3] Administrative records from health, education and social public services do not suffer from the same attrition biases by capturing all those receiving a service.[4] They are becoming increasingly available for research : initiatives in Wales and Scotland, have now created linked datasets derived from these data resources, and are using them to help direct local and national public health strategy.[5]

As yet, the potential gains from these 'big data' systems to drive local population-based analyses to improve child public mental health and educational services remain unrealised in England. Linkage of routinely collected data from public services has the potential to improve how local health, education and social care are delivered to children and young people. Certainly, all mental health services, hospital-based child health services, schools and child protection services which serve the same local area could be more efficient if the design, monitoring, targeting and integration of services were based on data.[6] The ethical and legal processes to do this, as well as the technical security requirements, to gain exemption from individual consent for health data are stringent.[7] Even once these challenges have been met, data matching processes can introduce challenges for health service researchers. For example, the introduction of bias by missed matches, particularly if risk factors are both associated with missed matched records and important outcomes, can impact the validity of research findings derived from linked data.[8] This is more likely to occur when linking routinely collected data via deterministic linkage approaches without a shared identification number (such as health and education records).[9] Deterministic linkage describes an approach when a set of predetermined rules are used to classify pairs of records as matched or nonmatched.These tend to require an exact or partial agreement on a set of personal identifiers for example a successful match on the first name or surname, and match on both the date of birth and postcode. Strict deterministic

For peer review only

For peer review only - http://bmjopen.bmj.com/site/about/guidelines.xhtml

methods are straightforward to use and commonly employed in government departments, however they can create high levels of missed matches between records.[10] As a consequence, this undermines the confidence that all the relevant records for an individual have been accurately combined across the different data sources.

In this study we show how an individual National Health Service (NHS) trust, with coverage of a geographically defined catchment of 1.2 million, ~190,000 children and young people (South London and Maudsley NHS Foundation Trust, SLaM) developed a sustainable approach to link and anonymise individual children and young people's records from healthcare, social and educational services. We show how a linkage environment that conformed to NHS and Department for Education (DfE) safeguards was used to build a data resource between a NHS child and adolescent mental health service (CAMHs) records via Clinical Record Interactive Search (CRIS) system[11] linked to the DfE's National Pupil Database (NPD).[12]

This study had two aims: the first was to provide a narrative description of the challenges in gaining approval for a research protocol which needed to meet the legislative requirements for both section 251 of the NHS Act 2006, via recommendation from NHS Health Research Authority Confidentiality Advisory Groups,[7] and The Education (Individual Pupil Information) (Prescribed Persons) (England) Regulations 2009[13] and subsequent amendments[14] - also demonstrating how the legal basis for the 'public benefit' can be made to satisfy General Data Protection Regulations (GDPR).[15] A second aim was to identify the socio-demographic and clinical factors risk factors, within a NHS CAMHS cohort, that were associated with non-matching with DfE educational records. As an applied example , we used the linked data resource to examine how non-matching may have impacted potential associations between child health factors and school absence (i.e. a key education outcome), and how statistical approaches could reduce the effects of this bias.

METHODS

The data resources

NHS Child and Adolescent Mental Health Service Data

SLaM provides comprehensive CAMHS to a geographic catchment of approximately 190,000 children and young people resident within four South London boroughs— Croydon, Lambeth,

For peer review only - http://bmjopen.bmj.com/site/about/guidelines.xhtml

Lewisham and Southwark. SLaM also provide highly specialist services which also accept referrals resident outside the four-borough catchment area. Clinical records have been fully electronic across SLaM services since 2007. The process by which CRIS permits these data to be available for research has been described in detail elsewhere.[6,11,16,17] In brief, CRIS extracts information from the electronic health records generated by CAMH services, and by removing personal identifiers, makes pseudo-anonymised data extracts available for analysis by SLaM approved researchers.

CRIS was used to provide an extract of children and young people who were referred to SLaM CAMHS services between 1st September 2007 and August 2013. SLaM has dedicated multidisciplinary services, which assess and treat school age children and young people under ICD-10 multi-axial classification system.[18] The tables and figures within the supplementary material describe the clinical sample by age and gender first accepted into SLaM CAMHS over a 5-year period. (supplementary table 1 for ICD-10 rates in the clinical sample). As supplementary figure 1-2 shows, the majority of children and young people are first seen in CAMHS services in mid-childhood and will often receive short discreet periods of care. However, some will receive prolonged CAMH services throughout child and adolescence.

Department for Education National Pupil Database

The NPD is a pupil level longitudinal database that matches pupil and school characteristic data to pupil level attainment.[12] The key datasets within the NPD are the pupil census and pupil attainment datasets, which holds data for all assessments that pupils complete during primary and secondary school state education. The census is a snapshot of pupils attending state-maintained schools in England ~ 91% of pupils resident with the SLaM catchment,[19] which is submitted annually on a specific day in January, by a school for all pupils in that school. Pupils held within the NPD are typically aged between 3-19 years, but some from special schools may be up to age 24.

The technical resources

To link CRIS data with other external clinical and non-clinical sources, SLaM developed a research governance model for linking data which satisfies NHS requirements as described in Department of Health Information Governance Review, or 'Caldicott 2' report.[20] In accordance with these guidelines, SLaM set-up the Confidential Data Linkage Service (SLaM CDLS)[11] as a Trusted Third Party or Safe Haven to ensure that confidential patient information can be linked in a way that guarantees the legal and ethical rights of patients and caregivers. A similar

For peer review only - http://bmjopen.bmj.com/site/about/guidelines.xhtml

provision was available in DfE Data Services Provision which had a linkage service, governed under HMG Security Policy Framework v10 2013 (SPF),[21] with experience of regularly undertaking external linkages with large scale research cohorts including the Millennium Cohort Study[22] and ALSPAC.[1]

Linkage

Preparing the CRIS CAMHS identifiers for matching.

We selected a cohort of young people aged between 4 and 18 years, who were referred to SLaM mental health care between 1st September 2007 and 31st December 2013. As described previously, in the UK, unique identifiers, such as national health identifiers, are not shared between health and education databases, so records require matching on personal identifiers common to both data resources (i.e. names, dates of birth, and residence post code).

Personal identifiers were standardised using the following definitions:

1. **Dob**: format (dd-mm-yyyy)
2. **forename_1**: The first word present in the forename field registered for the individual record. (i.e. all text left of the first white space character in the free text field)
3. **forename_2**: The second word present, if >1 forename present (i.e. second of 2+ names separated by one space or punctuation except "-") (i.e. right of white space)
4. **surname_1**: The first word present in the surname field registered for the individual record. (i.e. all text left of the first white space character)
5. **surname_2**: The second word present, if >1 Surname present (i.e. second word of 2+ names if separated by one space or punctuation except"-")
6. **surname_3**: The whole string in the surname field

Within the longitudinal health record, there were often several different addresses held for each individual. Similarly, there were multiple addresses held for most pupils in the education database. Pupil address data are routinely updated on the 16th January every year. So, we developed a hierarchical system to extract the postcode from the health record most likely to match with education database. Figure 1 shows how this postcode hierarchy might be applied to one individual child, where the blue blocks represent episodes of care provided by CAMHS, and the green time line represents the period of time in school. Taking these considerations

For peer review only

into account we produced a hierarchy of postcodes with 1 to 5 levels for each individual seen in CAMHS using logic rules (see figure 1 legend).

A SQL based query was used to extract the identifier data according to these rules. This produced a sample of 36,760 individuals with distinct individual records. Post extraction, we then ran data cleaning and logic checks which included removal of all those with numbers in name string fields (4 cases removed), all those with only one letter in their first or surname (1 case removed), all those with incomplete / atypical English postcodes (214 records hand searched, 77 valid English postcodes were cleaned and retained). We excluded all children whose first referral date was less than 4 years (1095 days) after their Date of birth, unless they had confirmed follow up contact details recorded within the window (i.e. 2007-2013) at least one year later than the earliest referral date. This was because clinicians can erroneously record the date of referral or time seen at initial appointment in the date of birth field. This mainly occurs in individuals with only single episodes of contact with services. To fit in with the academic calendar and UK school age, children were then selected if they had their 4th birthday prior to the 1st September 2012. This provided a complete sample of 35,509 ready for matching with the NPD.

All the data prepared for matching had personal identifier fields populated with the exception of the secondary surnames and forenames (i.e. there were no missing values). Dates of Birth ranged from 06/01/1989 – 31/08/2008 which meant that all of these pupils could potentially be found in either current or historic NPD census data. Personal identifiers were standardised to maintain a consistent format with NPD identifiers: SLaM identifiers were prepared to fit with DfE first name, surname and date of birth formats, which included standardising string length, capitalizations, use of spaces, and hyphens.

Only identifiers (names, postcode and date of birth), accompanied by their unique CRIS ID pseudonym, were then sent via secure file transfer to the DfE Data and Statistics Department.

As represented in figure 2 (and described in four stages below) , the DfE matched these against NPD personal identifiers (approximately 15 million records), generating a pupil-specific, non-identifiable NPD ID variable across the whole data set, and adding the CRIS ID to this table for cases only, stripping the resultant table of all identifiers other than the anonymised NPD ID and the pseudonymised CRIS ID, and transferring the data set back to SLaM CDLS using a secure file transfer.

For peer review only

The supplied data items by the SLaM CDLS were matched to the NPD data by DfE informaticians in the stages described below. Initial matching or stage 1 was based on exact matches for the supplied data items. For those cases who did not match at stage 1, stage 2, 'fuzzy' matching processes were conducted, and so on, down to stage 4.

- Stage 1: Full match on any combination of CAMHS names (all supplied values including alias), dates of birth and postcode (all supplied) were conducted against the most recent address held, and then the working back, all years/terms of the School census data, Pupil Referral Data, Alternative Provision Data, Early Years Census data. School census data contained preferred and former surnames, which were also searched. Forenames were checked against forename/middle name combinations.

- Stage 2: Full match on Date of Birth, Postcode and Fuzzy matching on names. To ensure confidence in these matches, results were checked manually. Fuzzy matching was conducted on first two characters of names**.**

- Stage 3: Full match on names and dates of birth, postcode inward code (the first 2-4 characters) plus first character of the outward code (the latter characters after the space). To ensure confidence in these matches, results were checked manually.

- Stage 4: Full match on names and Postcode with manual check of dates of birth, looking for 'near' dates of birth – where the record may be possibly one year out, one month out, one day out, and transposed month/day.

7.3.4 Analysis of linkage bias

Overall linkage rate was calculated as the percentage of CAMHS individuals linked to any NPD school record on any of the stages 1-4. Potential sources of linkage biases were estimated by comparing linked and unlinked data. For the CAMHS sample described in table 1, we categorised an individual match to NPD school absence data (a subset of the NPD school record) as a binary outcome: match =1, non-match=0. The ICD-10 multi-axial classification system[18] was used to categorise the presence of any recorded mental health diagnosis (i.e. diagnoses status prior to 18th birthday) available between 2007 and 2013.

Using multivariable logistic regression, we explored the associations between a number of risk variables including demographic (e.g. gender, ethnicity, neighbourhood deprivation), clinical

For peer review only - http://bmjopen.bmj.com/site/about/guidelines.xhtml

(age at first presentation to CAMHS, diagnosis of any ICD-10 disorder) and administrative factors (e.g. postcode hierarchy, see Figure 1) with linkage to the school attendance database as the binary outcome. We used this logistic regression to generate a probability estimate of matching as a function of the risk variables.

Patient and public involvement

In terms of gathering evidence for support of the public benefit to use patient identifiable data via CRIS to link to the national pupil database without patient or caregiver consent, we consulted several clinical, patient and caregiver groups. We invited comments on privacy notices, gave presentations, and collected minutes from the SLaM child and adolescent psychiatry executive group, the Service User Research Enterprise group (SURE), National Young Persons Advisory Group, the service user led CRIS Oversight Committee, and SLaM-involved parents, through the BRC patient engagement programme.[23] Because of the focus of one of the projects using the linked data was an investigation into the educational outcomes of children and young people with Autism Spectrum Disorders, we also invited comments on the proposal from the National Autistic Society. A lay summary of the purpose of the data linkage was written in collaboration with the Maudsley NIHR service user data linkage advisory group (eg, https://www.maudsleybrc.nihr.ac.uk/facilities/clinical-record-interactive-search-cris/cris-data-linkages/) and a short video was made, to raise awareness of the study and future research plans.

Analysis of linkage error using school attendance outcomes

School absence was chosen as the outcome to assess linkage error because is it challenging to assess the impact of the error for a particular outcome, when there is not an expected one-to-one relationship between one variable and another. For example, when linking patient records to a death registry to determine a patient's survival status, it is difficult to know which matches have been missed – the death registry will only contain patients who have died, and so a non-match could be due to patient being alive or being a missed match.[24] Applying this to school data, there was a need to select a clinically relevant school performance outcome which should be available for all pupils. School attendance should be recorded for all pupils accessing state school, and was clinically relevant, hence useful as outcome for evaluating the impact of linkage error.

For peer review only - http://bmjopen.bmj.com/site/about/guidelines.xhtml

For each matched CAMHS-NPD pupil, a binary outcome marker of poor attendance was created for the latest academic year they attended school available between 2007/08 and 2012/13. Pupils were categorised as persistent absentees if they had recorded less than 80% school attendance for the total number of possible school sessions available since their enrolment for that academic year (one session is equal to half a school day).

Using the probability of matching estimate from the linkage bias analysis, we created a weight that was inversely proportional to the probability of being linked to national pupil database school attendance data, which was assigned to each individual with linked CAMHS-school absence data. This followed standard methodology for managing non-response bias in conventional cohort and survey designs.[25] Multivariable logistic regression was used to examine predictor variables and association with persistent school absence, initially without weights, and then with inverse probability weights. To examine another approach to adjust for potential selection bias from non-linkage,[26] we examined whether the main effects of interest also persisted after the probability of matching estimate was entered as a covariate in the multivariable logistic regression model.

RESULTS

Section 1: Achieving the ethical, governance and legal approvals

The proposal to link the NPD and CRIS CAMHS data, underwent a robust and lengthy ethical, legal, governance and technical review, conducted by a number of local and national committees within NHS and DfE. Figure 3 provides the timeline and milestones achieved to reach the completion of the linked DfE-SLaM CAMHS dataset. We provide in depth description of the process as a supplementary report to this paper. In brief, gaining the permissions to link the NPD and CRIS CAMHS data was complex, as there was no precedent in England for such a linkage between routinely collected mental health and school data, and there had been no successful completion of linked NHS and non-NHS non health data without individual consent.[27] After a round of discussions between DfE and SLaM, we described a process to link the data, with the main research purpose focused on estimating the effects of clinically recognised, mental health disorder and treatment on educational outcomes. Research Governance approval was granted by the SLaM Caldicott Guardian Committee and the DfE's

For peer review only

Data Management Advisory Panel in principle, but the linkage process was contingent on Health Research Authority Confidentiality Advisory Group (HRA CAG) approval.[7]

The HRA CAG rejected the first application, as the research activity proposed did not demonstrate sufficient medical purpose and public benefit to meet the s251 requirements (please see supplementary report for further details). Research conducting a longitudinal analyses of health exposures on education outcomes was not sufficient to meet criteria for conducting research for medical purpose. The HRA CAG also queried whether linkages could not be better carried out using NHS Digital's Trusted Data Linkage Service. The CAG advised that this route would negate the requirement for SLaM to disclose confidential patient information to the DfE, and minimise the disclosure of patient information. A final major issue related to the governance arrangements in place around the processing of patient data by the DfE. We hadn't provided sufficient information around retention periods, access arrangements and the extent of identifiable data requested. To prepare for resubmission, we revised the scientific proposal to be more focused on understanding the bi-directional associations between educational performance and mental health disorders. To gather more evidence the public benefit case of scientific proposal, we involved our local NIHR Biomedical Research Centre patient and clinician engagement programme, relevant charitable and education sector bodies.

To address the second issue, we acknowledged that an additional potential benefit to using NHS Digital was that patient identifiers would be retained within a NHS environment, but we were able to confirm that both the SLaM CDLS and DfE were in line with government standards and meet equivalent to information governance (IG) expectations for NHS care system organisations. [28]We also demonstrated, by reviewing the alternative data flows, that using NHS Digital as the trusted third party in this linkage would prove a more complex, and less secure linkage method (please see supplementary report). Briefly, both DfE and SLaM data controllers expressed concern that the additional step of involving NHS Digital, would significantly increase the potential risk of harm if a breach of data security occurred, especially given the scale and sensitivity of the educational data, and the very large number of individuals involved (over 15 million children).

Section 2: Linkage rates, bias and the impact on education outcome analyses

The overall matching process against any National Pupil Database attendance records provide 29,278 CAMHS-NPD linked records representing a linkage rate of 82.5%. The proportions linked according to DfE matching stages described above: stage 1 - 60.2%; stage 2 - 4.2%; Stage 3 – 1.2% and Stage 4 – 16.9%.

For peer review only - http://bmjopen.bmj.com/site/about/guidelines.xhtml

Table 1 identifies the SLaM CAMHS socio-demographic, clinical and administrative record risk factors for linkage to the NPD data. An odds ratio greater than 1 denotes greater chance of successful linkage compared to the reference. In the adjusted model, we found significant differences in most socio-demographic, clinical and administrative factors. Compared to school age children aged under 7, children first referred to CAMHS in late adolescence were significantly less likely to be matched to the NPD (OR 0.67, 95% C.I. 0.59-0.75, $p<0.01$), whilst children aged between 7 and 12, were more likely to be successfully matched (OR 1.23, 95% C.I. 1.10-1.38, $p<0.01$). Relative to children of White ethnicity, we found other ethnic groups including Asian, Black African and Mixed groups were less likely to be matched. There were no significant differences in successful linkage between children and young people in the lowest and highest quartiles of deprivation, but there was significantly reduced linkage success for those living in neighbourhoods in the 2nd and 3rd quartiles. Analyses of the administrative characteristics show that the post codes (which were extracted from clinical episodes of care and did not overlap with January census data (i.e. post codes 2,4 and 5, see figure 1) were less likely to link even after adjustment for other potential explanatory variables (see table 1).

Table 2 provides the socio-demographic, clinical and administrative record characteristics for children and young people seen SLaM CAMHS and the associated risk for persistent absence. The adjusted analyses show that presence of an ICD-10 mental health disorder (aO.R 1.13, 95% C.I. 1.07-1.22, $p <0.01$), age at first referral to CAMHS and Mixed ethnic group (relative to white ethnic groups), were associated with an increased risk of persistent school absence, whilst Asian, Black African, Black Caribbean ethnicity, increased neighbourhood affluence was associated with a decreased risk of persistent absence. These effects persisted after both statistical techniques i) using inverse probability weighting, and ii) adjustment for matching probability were applied to reduce matching bias in the adjusted analyses.

DISCUSSION

We provide the first example of how data linkage projects can be completed using routinely collected NHS and DfE Educational data. This use case demonstrates how the legal basis for the 'public benefit' (i.e. without individual level consent) can be made to satisfy General Data Protection Regulations (GDPR).[15] The regulatory and technical issues for data sharing between health and non-health services are challenging in England, but surmountable. Using deterministic matching techniques provided by the DfE, a large-scale dataset was built between NHS child and mental health data and national school administrative data, providing a linkage

For peer review only

for 29,278 patients (82.5% of the NHS cohort) to their educational records. There were significant differences in the socio-demographic and clinical characteristics between matched and non-matched NHS samples. Using these data, we found any child or young person with a ICD-10 mental disorder had approximately 10% greater likelihood of having persistent school absence, when compared to those clinically referred and not meeting threshold for diagnosis. Effects did not change significantly after matching probability adjustment, which suggests these effects on were not driven by selection bias from matching errors.

Analysis of the linkage biases

Overall, we found 17.5% of the clinical population were not successfully matched to NPD absence data. Whilst enrolment at a non-state maintained school or independent school may explain a proportion,[6,19] a significant minority were likely to match due to administrative factors, which may include missingness or inconsistencies of the matching identifiers, as demonstrated by the effect of post code variation in the analysis, or errors secondary to the matching process. There have been very few studies conducted which examine linkage errors in children and young people, especially where the non-linked group are not subject to consent related bias. Previous research suggests that ethnic minorities are more likely to have administrative records with misspelt names, inaccurately recorded dates of births, and higher levels of residential instability, which may be applicable to this sample.[9,29] These findings provide further argument for greater collaborative research with data providers to develop linkage methods that minimize potential biases in analyses of linked data. [10] Deterministic process which offers little flexibility in matching misspelt names may be a reason why ethnic variation may contribute to missed matches.[9] We found certain age groups, particularly those aged 7 to 12, were associated with a greater likelihood of linkage. This may be due to the greater availability of accurate personal identifiers in the records of this group, as their potential exposure to CAMHS services whilst at school will be longer than other age groups. Similarly, having a ICD-10 mental disorder, which also had an increased likelihood of linking with the school data, may be related to identifier accuracy, as their higher levels of psychopathology will be associated with greater clinical contact, and potentially higher clerical accuracy in recording personal identifiers. It is also more probable that those with higher levels of psychopathology will have longer durations of care that overlap with the school census date.

We found a U-shaped distribution in neighbourhood deprivation and likelihood of linkage. Compared to areas with the highest deprivation, areas within the 2nd and 3rd quartiles showed

For peer review only

significantly reduced likelihood of linkage, but the most affluent areas showed minimal difference. This could relate to families from affluent areas being able to comply with the administrative process, and/or correct administrative errors, and families from the highest deprived areas having greater need and hence higher clinical contact with services. Both these factors may improve clerical accuracy and concordance with school data. Families from 2nd and 3rd quartiles may have less of both these characteristics, and hence reduce their likelihood of linkage.The current data available in this study does not permit this hypothesis to be tested, but findings suggest that a more detailed extraction examining frequency of clinical contact with services and data linkage outcome is an area for future work.

In our sample, linkage biases appear to have little effect on the association between mental disorder and attendance. However, without information from source data, potential linkage error could be introduced without researchers being aware whether there was need for it to be accounted for in subsequent analyses. Our study highlights the importance of governance arrangements between linkers and analysts to identify which groups are disproportionately affected by linkage error. In our case, by permitting approved NHS researches to examine the identifier fields of matched and unmatched SLaM samples, this governance has enabled some flexibility with the 'data separation principle' : a common practice in data linkage research, where identifiers (e.g. names or date of birth) are kept separate from attributes (in this case health or education data), to protect privacy and avoid disclosure during the linkage process.[30] While the separation principle might reduce the risk of identification, it does not permit researchers to evaluate the potential risk of linkage bias on future analyses.

Implementation challenges to the data linkage between health and education data

We believe the tasks and challenges to use personal health and education data for data linkage and research can be best described as 'establishing the social license'.[31] This activity included articulating a clear purpose for the linkage, recognized as beneficial by the public or those potentially involved as data subjects, and that the potential risks to individuals or public institutions were tolerable in relation to these benefits. Without the evidence of the proposal being scrutinized and ultimately accepted by those potentially involved as data subjects, and the public institutions/services who act as controllers of the data, it would have been difficult to sustain a case for public benefit – in fact this was one of the reasons why the first application was not approved by the HRA CAG. To prove we had *social licence* to conduct the linkage work, we needed to gather evidence from a number of sources including service users,

For peer review only - http://bmjopen.bmj.com/site/about/guidelines.xhtml

clinicians, academics, advocacy groups, governance leads; all who may have had a stake in the process and outcomes of the data linkage project.

The second aspect of establishing a social licence, related to fulfilling the professional mandate for properly conducting the linkage process and related research activity. This involved making sure the proposal complied with the known legal, technical and ethical frameworks that governed health data use, and any additional safeguards deemed important by the data controllers and custodians. The technical aspects were not just confined to data security, but also involved preparing the data to ensure the most accurate match, to reduce error and redundancy in later analysis. Fulfilling the mandate also involved the creation of formal contract between the parties involved in controlling, sharing, processing and using the data. This mandate committed us to conduct appropriate analysis and dissemination of the linkage related research, so that we could sustain the social license for future research activity. This may be especially pertinent in England as linkage driven research of routinely collected public service activity is in its infancy, and benefits are yet to be comprehensively established.

Given the time and resources spent to set up this linked data resource, and the potential it holds, it is important that these resources are maintained, and remain accessible for re-use in the future. Without developing specific data sharing agreements between the parties, it can be difficult to establish a collaborative relationship with good governance structures between the controllers, linkers and analysts. Without these structures, there may be a tendency for data controllers to agree to link data only via a 'create and destroy' approach. We believe this maybe unethical in terms of waste and scientifically unsound as prior analyses cannot be re-examined. It also re-exposures data subjects to the potential risks of sharing personal identifiable information again across different agencies should the linkage need to be repeated in the future.

Strengths and Limitations of the matching methods and matching evaluation

This study has a number of strengths. First it presents a novel application to link data across public sector organisations. The description of the legal, ethical and technical challenges and solutions are described to share some of the lessons we have learned through the process, in the hope that they will be useful for other public organisations. Furthermore, the study provides an example of how potential non-random loss between routinely collected health and non-health linked data can be adjusted by weighting techniques. Because the source data was available to examine missed linkages, we were able to determine that linkage error did not lead to

For peer review only

systematic biases and misleading positive estimates between ICD-10 mental disorder and persistent school absence. The demonstration of matching probability adjustment and inverse probability weighting was intended to illustrate how linkage bias may be reduced, not as a definitive analysis of these data. Given its policy relevance, we reported on a single categorical absence outcome, less than 80% annual school attendance. Whether the same associations hold for other discrete levels of absence (e.g. 60% or 90%) certainly warrants examination in future analyses. Examining methods to improve linkage techniques, coupled with newer methods for handling uncertainty in analysis of linked data, should also help improve the generalisability and quality of future population-based linkage studies.[27]

The matching methods in this study have a number of limitations. We were unable to assess false positive matching, nor able to assess risks for the lower confidence matching (DfE stages 2-4, described above), and the potential effects on school outcome analyses. No shared unique identifier exists between NHS and educational services, nor were their governance arrangements or sufficient resources in place to manually compile a NPD-SLaM CAMHS linked gold-standard data. Another limitation of the matching methodology is the limited number of address identifiers that could be used. For example, due to governance constraints we were unable to use first line of the address, which again limited the capacity to potentially check for coding errors in the postcode. Another contributing factor to linkage error was the age of the child. A substantial number of young people were seen in CAMHS aged 16 and 17 years and would not have data on the NPD if they were no longer attending school. Similarly, we were unable to determine who was not eligible for matching due to complete private or home school educational provision which may be, at greatest, 10% of the sample. These limitations are likely to have a led to our finding being an underestimation of the linkage performance.

The matching evaluation also has several limitations. We only reported on a single categorical absence outcome (less than 80% annual school attendance); whether linkage error had similarly limited effects on other discrete levels of absence (e.g. 60% or 90%) was not evaluated. ICD-10 codes permitted us to evaluate the effect of reaching threshold for a “clinical disorder” on absence rates in an efficient and cost-effective manner. However, collapsing ICD-10 categories into one binary variable only provided an ‘average’ effect across all ICD-10 diagnoses. This may have introduced aggregation bias, which disguised the potential heterogeneity of effects across different the diagnoses. Furthermore, the validity of ICD-10 codes in psychiatric registers can be variable, and although we did not disaggregate ICD-10 cases into specific disorders, it is known some disorder codes are more likely to be misclassified than others, or at least more prone to diagnostic revision.[32] Assessing the effect of variation in ICD-10 validity

For peer review only - http://bmjopen.bmj.com/site/about/guidelines.xhtml

on school outcomes was beyond the scope of this study. However, we have provided solid ground-work for future research to refine the characterisation of clinical phenotypes either via algorithms that offer greater diagnostic precision for case-ascertainment (such as an ICD-10 twice coding rule[33]) or take advantage of computational linguistic techniques (e.g. free-text extraction using natural languages processing approaches).[11,34]

Implications

The work described sets a precedent for education data being used for patient or medical benefit in England. The regulatory and technical issues for data sharing between health and non-health services are challenging. Certainly, to develop and improve linked data resources, partnerships between academic and government institutions should continue to explore public opinion and develop guidance on building a 'social license' for the sustained use of linked data.[31] In addition, it is important that recent policies which support accessibility for re-use in the future are sustained, especially given the time and resources spent to set up linked data resources, and the potential they hold.[35]

Record linkages are a valuable enhancement to child-based longitudinal studies and clinical registries, which allow evaluation of questions relevant to public health and social care policy. We would urge all mental health trials conducted in children that might influence their attendance or function at school to link to the National Pupil Database. We hope our experience may provide a useful guide for other health services wishing to build information resources using linked administrative data, and specifically to encourage other mental health service providers to work together to link their data to National Pupil Database. In time we hope these resources will generate a wider network of fine-grained data and analytical expertise, which can be used for research to inform commissioning and service provision and better meet children and young person's mental health needs within the population.

Footnotes

- Contributors: The study was conceived by JD, TF and MH. Data extraction was carried out by JD with support from HS, MB, SE, RL and AJ. Data analysis was undertaken by JD. Reporting of findings was led by JD with support from RG, TM, SE, TF, JDe and RH, supervised by RS and MH. All authors contributed to manuscript preparation and approved the final version.

For peer review only - http://bmjopen.bmj.com/site/about/guidelines.xhtml

- Role of Funding Source. This work was supported by the Clinical Records Interactive Search (CRIS) system funded and developed by the National Institute for Health Research (NIHR) Mental Health Biomedical Research Centre at South London and Maudsley NHS Foundation Trust and King's College London and a joint infrastructure grant from Guy's and St Thomas' Charity and the Maudsley Charity (grant number BRC-2011-10035). J.D. received supported by a Medical Research Council (MRC) Clinical Research Training Fellowship (MR/L017105/1) and Psychiatry Research Trust Peggy Pollak Research Fellowship in Developmental Psychiatry. RDH was funded by a Medical Research Council (MRC) Population Health Scientist Fellowship (grant number MR/J01219X/1). MH, RS, AS, MB, RL, HS received salary support from the National Institute for Health Research (NIHR) Mental Health Biomedical Research Centre at South London and Maudsley NHS Foundation Trust and King's College London. JDe was supported by the National Institute for Health Research (NIHR) Collaboration for Leadership in Applied Health Research and Care North Thames at Bart's Health NHS Trust (NIHR CLAHRC North Thames). RG and JDe are members of the Policy Research Unit in the Health of Children, Young People and Families (CPRU), which is funded by the England Department of Health Policy Research Programme. The views expressed are those of the author(s) and not necessarily those of the NHS, the NIHR or the Department of Health and Social Care.
- Competing Interests : None declared
- Ethics approval: The CRIS data resource received ethical approval as an anonymised data set for secondary analyses from Oxfordshire REC C (Ref: 08/H0606/71+5); and NHS Health Research Authority Confidentiality Advisory Group, reference: CAG 9-08(a)/2014.
- Data sharing statement The data accessed by CRIS remain within an NHS firewall and governance is provided by a patient-led oversight committee. Subject to these conditions, data access is encouraged and those interested should contact RS (robert.stewart@kcl.ac.uk), CRIS academic lead.

For peer review only

Additional files:

Figure 1: Creating a hierarchy of matching postcodes to improve the link between CRIS CAMHS Data to DfE National Pupil Database

Figure 2: Data flow process linking CRIS CAMHS Data to the National Pupil Database

Figure 3: A timeline of the ethical, legal and technical milestones for reaching a data linkage between DfE and SLaM

Supplementary file 1:

1) Report on providing a linked health and educational data resource: achieving the ethical, governance and legal approvals
2) Supplementary Figure 1: Number of accepted first referrals for all children and young people (aged 4 -17) seen by SLaM CAMHS services (Sept 2007 – August 2013)
3) Supplementary Figure 2: Duration between first and last contact with mental health professionals for children and young people (aged 4 -17) accepted to SLAM CAMHS between Sept 2007 – August 2013.
4) Supplementary table 1: Diagnostic breakdown of all children (aged 4 -17) referred to SLaM CAMHS services between Sept 2007 and August 2013.

REFERENCES

1 Overy C, Reynolds LA, Tansey EM. History of the Avon Longitudinal Study of Parents and Children, C 1980-2000. Queen Mary, University of London, 2012 http://qmro.qmul.ac.uk/xmlui/handle/123456789/2827 (accessed Sept 22, 2018).

2 Bonevski B, Randell M, Paul C, *et al.* Reaching the hard-to-reach: a systematic review of strategies for improving health and medical research with socially disadvantaged groups. *BMC Med Res Methodol* 2014; **14**: 42.

3 Kho ME, Duffett M, Willison DJ, Cook DJ, Brouwers MC. Written informed consent and selection bias in observational studies using medical records: systematic review. *BMJ* 2009; **338**: b866.

4 Langan SM, Benchimol EI, Guttmann A, *et al.* Setting the RECORD straight: developing a guideline for the REporting of studies Conducted using Observational Routinely collected Data. *Clin Epidemiol* 2013; **5**: 29–31.

5 Public Health Research Data Forum. Enabling Data Linkage to Maximise the Value of Public Health Research Data: full report. Wellcome Trust, 2015 https://wellcome.ac.uk/sites/default/files/enabling-data-linkage-to-maximise-value-of-public-health-research-data-phrdf-mar15.pdf (accessed Sept 22, 2018).

6 Downs J, Gilbert R, Hayes RD, Hotopf M, Ford T. Linking health and education data to plan and evaluate services for children. *Arch Dis Child* 2017; **102**: 599–602.

7 Health Research Authority. Section 251 and the Confidentiality Advisory Group (CAG). Health Res. Auth. http://www.hra.nhs.uk/about-the-hra/our-committees/section-251/ (accessed Sept 21, 2018).

8 Lariscy JT. Differential Record Linkage by Hispanic Ethnicity and Age in Linked Mortality Studies Implications for the Epidemiologic Paradox. *J Aging Health* 2011; **23**: 1263–84.

For peer review only

9 Bohensky M. Bias in data linkage studies. In: Harron K, Goldstein H, Dibben C, eds. Methodological Developments in Data Linkage. John Wiley & Sons, Ltd, 2015: 63–82.

10 Harron K, Goldstein H, Dibben C. Methodological Developments in Data Linkage. Chichester, West Sussex, United Kingdom: Wiley-Blackwell, 2015.

11 Perera G, Broadbent M, Callard F, *et al.* Cohort profile of the South London and Maudsley NHS Foundation Trust Biomedical Research Centre (SLaM BRC) Case Register: current status and recent enhancement of an Electronic Mental Health Record-derived data resource. *BMJ Open* 2016; **6**: e008721.

12 Department for Education. National Pupil Database User Guide. UK Government, 2015 https://www.gov.uk/government/uploads/system/uploads/attachment_data/file/261189/NPD_User_Guide.pdf (accessed Jan 3, 2015).

13 The Education (Individual Pupil Information) (Prescribed Persons) (England) Regulations 2009. http://www.legislation.gov.uk/uksi/2009/1563/contents/made (accessed Sept 21 ,2018).

14 The Education (Individual Pupil Information) (Prescribed Persons) (England) (Amendment) Regulations 2013. http://www.legislation.gov.uk/uksi/2013/1193/made (accessed Sept 21 , 2018).

15 Information Commissioner's Office. Guide to the General Data Protection Regulation. 2018 https://ico.org.uk/media/for-organisations/guide-to-the-general-data-protection-regulation-gdpr-1-0.pdf (accessed May 20, 2018).

16 Downs J, Hotopf M, Ford T, *et al.* Clinical predictors of antipsychotic use in children and adolescents with autism spectrum disorders: a historical open cohort study using electronic health records. *Eur Child Adolesc Psychiatry* 2016; **25**: 649–58.

17 Downs JM, Lechler S, Dean H, *et al.* The Association Between Comorbid Autism Spectrum Disorders and Antipsychotic Treatment Failure in Early-Onset Psychosis: A Historical Cohort Study Using Electronic Health Records. *J Clin Psychiatry* 2017; **78**: e1233–41.

18 Rutter M, World Health Organization. Multiaxial classification of child and adolescent psychiatric disorders : the ICD-10 classification of mental and behavioural disorders in children and adolescents. Cambridge : Cambridge University Press. Cambridge: Cambridge University Press, 1996.

19 Department for Education. London Schools and Pupils by Borough and Type of School. 2015 https://data.london.gov.uk/dataset/schools-and-pupils-type-school-borough (accessed May 22, 2018).

20 The Information Governance Review. Information: To share or not to share? Department of Health, UK Government, 2013 https://www.gov.uk/government/uploads/system/uploads/attachment_data/file/192572/2900774_InfoGovernance_accv2.pdf (accessed May 7, 2017).

21 HMG Cabinet Office. Security policy framework. 2016. https://www.gov.uk/government/publications/security-policy-framework (accessed 21 Sept 2018, 2018).

22 Welcome to the Millennium Cohort Study - Centre for Longitudinal Studies. https://www.cls.ioe.ac.uk/page.aspx?sitesectionid=851 (accessed March 26, 2018).

For peer review only

23 King's College London - Service User Research Enterprise (SURE). https://www.kcl.ac.uk/ioppn/depts/hspr/research/ciemh/sure/SURE.aspx (accessed May 22, 2018).

24 Bohensky MA, Jolley D, Sundararajan V, *et al.* Data Linkage: A powerful research tool with potential problems. *BMC Health Serv Res* 2010; **10**: 346.

25 Höfler M, Pfister H, Lieb R, Wittchen H-U. The use of weights to account for non-response and drop-out. *Soc Psychiatry Psychiatr Epidemiol* 2005; **40**: 291–9.

26 Little RJ, Vartivarian S. On weighting the rates in non-response weights. *Stat Med* 2003; **22**: 1589–99.

27 Health Research Authority. CAG Advice and HRA/SofS Approval Decisions. Health Res. Auth. https://www.hra.nhs.uk/planning-and-improving-research/application-summaries/confidentiality-advisory-group-registers/ (accessed May 22, 2018).

28 Department for Health. NHS Information Governance Toolkit. https://www.igt.hscic.gov.uk/ (accessed Sept 21, 2018, 2018).

29 Hagger-Johnson G, Harron K, Goldstein H, Aldridge R, Gilbert R. Probabilistic linkage to enhance deterministic algorithms and reduce data linkage errors in hospital administrative data. *J Innov Health Inform* 2017; **24**: 234–46.

30 Gilbert R, Lafferty R, Hagger-Johnson G, *et al.* GUILD: GUidance for Information about Linking Data sets. *J Public Health* 2018; **40**: 191–8.

31 Carter P, Laurie GT, Dixon-Woods M. The social licence for research: why care.data ran into trouble. *J Med Ethics* 2015; **41**: 404–9.

32 Davis KAS, Sudlow CLM, Hotopf M. Can mental health diagnoses in administrative data be used for research? A systematic review of the accuracy of routinely collected diagnoses. *BMC Psychiatry* 2016; **16**: 263.

33 Hebbring SJ. The challenges, advantages and future of phenome-wide association studies. *Immunology* 2014; **141**: 157–65.

34 Downs J, Dean H, Lechler S, *et al.* Negative Symptoms in Early-Onset Psychosis and Their Association With Antipsychotic Treatment Failure. *Schizophr Bull* 2018. DOI:10.1093/schbul/sbx197.

35 Administrative Data Research Network. Data Reuse for Research Purposes. Economic and Social Research Council, 2017 https://adrn.ac.uk/media/174422/adrn-034_datareuseforresearchpurposes_02_00_pub.pdf (accessed May 21, 2018).

For peer review only

Table 1: Socio-demographic characteristics of the Child and Adolescent Mental Health sample linked and non-linked to the national pupil database absence data

	Linked pairs (n=29,278)	Non-linked residuals (n=6,231)	O.R (95% C.I.) for +ve linkage	aO.R (95% C.I.)
Male	16,430 (56.1%)	3,296 (52.9)	*Reference*	*Reference*
Female	12,848 (43.9%)	2,935 (47.1)	0.88 (0.83-0.93)**	1.04 (0.97-1.11)
**Age at first referral to mental health services**				
Infant (<7yrs)	3657 (12.5%)	535 (8.7%)	*Reference*	*Reference*
Primary (7-12 yrs)	10,980 (37.5%)	1,284 (20.3%)	1.25 (1.12-1.39)**	1.23 (1.10-1.38)**
Secondary (13-15 yrs)	7,048 (24.1%)	1,140 (18.4%)	0.90 (0.81-1.01)	0.98 (0.88-1.10)
College (16-18)	7570 (25.9%)	3228 (52.2)	0.34 (0.31-0.38)**	0.67 (0.59-0.75)**
**Ethnicity**				
White / White-British	13,838 (47.3%)	2,786 (44.7)	*Reference*	*Reference*
Asian / Asian-British	984 (3.4%)	312 (5.0%)	0.63 (0.56-0.76)**	0.65 (0.56-0.75)**
Black British / African	5,667 (19.4%)	1,181(19.0%)	0.96 (0.89-1.04)	0.82 (0.76-0.89)**
Black British / Afro-Caribbean	1,474 (5.0%)	232 (3.7%)	1.28 (1.11-1.48)**	0.98 (0.84-1.14)
Mixed / Multiple ethnic	2,184 (7.5%)	315 (5.1%)	1.40 (1.23-1.58)**	1.12 (0.99-1.28)
Other ethnic group	1,109 (3.8%)	419 (6.7%)	0.53 (0.47-0.60)**	0.55 (0.48-0.63)**
Not stated	4,022 (13.7%)	986 (15.8%)	0.82 (0.76-0.89)**	0.93 (0.85-1.02)
**Resident within Local catchment area**	22,481 (76.8%)	4,192 (67.2%)	1.61 (1.52-1.71)**	1.04 (0.97-1.12)
**National quartiles of Neighbourhood deprivation**				
1st (Most deprived)	14,398 (49.2%)	2,822 (45.3%)	*Reference*	*Reference*
2nd	9,796 (33.5%)	2,179 (34.9%)	0.88 (0.83-0.94)**	0.90 (0.83-0.96)**
3rd	2,956 (10.1%)	762 (12.2%)	0.76 (0.69-0.83)**	0.81 (0.74-0.89)**
4th (Least Deprived)	2,126 (7.3%)	468 (7.5%)	0.89 (0.79-0.99)*	1.03 (0.92-1.15)
**Address data available2**				
Postcode 1	17,587 (60.1%)	1,987 (31.9%)	*Reference*	*Reference*
Postcode 2	2,956 (10.1%)	990 (15.9%)	0.34 (0.31-0.37)**	0.50 (0.45-0.56)**
Postcode 3	5,776 (19.7%)	1,187 (19.1%)	0.55 (0.51-0.59)**	0.63 (0.58-0.68)**
Postcode 4	1,933 (6.6%)	1,010 (16.2%)	0.22 (0.20-0.23)**	0.35 (0.31-0.39)**
Postcode 5	1,026 (3.5%)	1,057 (17.0%)	0.11 (0.09-0.12)**	0.15 (0.14-0.17)**
**Any ICD-10 Disorder**	17,749 (60.6%)	3,290 (52.8%)	1.38 (1.30-1.45)**	1.11 (1.04-1.18)**

*P < 0.05,** P <0.01
[1]adjusted for all other co-variates listed in the table.
[2] Post code. For a large proportion of cases there are several addresses available for each case. Therefore, postcodes were extracted according to a hierarchy (Postcode 1 being the highest) which we believed to be most likely to have been the place of residence on the day of the 16th Jan 20XX (variable date) census. [See Figure 1 legend]

For peer review only

Table 2: Socio-demographic and odds ratios for persistent (>80%) school absence in 29, 278 children and young people referred to mental health services

	No persistence Absence (n=23,241)	Persistent School Absence (n=5,635)	O.R (95% C.I.)	aO.R[1] (95% C.I.)	Weighted aOR[2]	Match probability adjusted aOR[3]
**Any ICD-10 Disorder**	14,004 (60.2%)	3,594 (63.7%)	1.16 (1.09-1.23)**	1.13 (1.07-1.22)**	1.13 (1.07-1.22)**	1.10 (1.03-1.19)**
**Age at first referral to mental health services**						
<7yrs	3,031 (13.0%)	298 (5.3%)	Reference	Reference	Reference	Reference
7-12 yrs	9,405 (40.5%)	1,540 (27.3%)	1.67 (1.46-1.90)**	1.67 (1.46-1.90)**	1.67 (1.47-1.91)**	1.60 (1.49-1.84)**
13-15 yrs	5,205 (22.4%)	1,830 (32.5%)	3.58 (3.14-4.07)**	3.65 (3.20-4.18)**	3.71 (3.24-4.23)**	3.66 (3.21-4.18)**
16-18 years	5,600 (24.1%)	1,967 (34.9)	3.57 (3.13-4.06)**	4.20 (3.63-4.86)**	4.15 (3.57-4.81)**	4.70 (3.82-5.78)**
**Female**	10,023 (43.1%)	2,695 (47.8%)	1.20 (1.14-1.28)**	0.97 (0.91-1.03)	0.97 (0.92-1.04)	0.96 (0.91-1.03)
**Ethnicity**						
White / White-British	10,651(45.8%)	3,011(53.4%)	Reference	Reference	Reference	Reference
Asian / Asian-British	815 (3.5%)	159 (2.8%)	0.69 (0.58-0.82)**	0.68 (0.57-0.81)**	0.69 (0.58-0.83)**	0.76 (0.60-0.96)*
Black British / African	4,737 (20.4%)	849 (15.1%)	0.63 (0.58-0.69)**	0.68 (0.62-0.74)**	0.69 (0.63-0.75)**	0.71 (0.64-0.79)**
Black British / Afro-Caribbean	1,213 (5.2%)	248 (4.4%)	0.72 (0.63-0.83)**	0.81 (0.70-0.94)**	0.81 (0.70-0.94)**	0.82 (0.70-0.94)**
Mixed / Multiple ethnic	1,653 (7.1%)	483 (8.6%)	1.03 (0.93-1.15)	1.14 (1.02-1.28)*	1.15 (1.03-1.29)*	1.11 (0.99-1.26)
Other ethnic group	905 (3.9%)	195 (3.5%)	0.76 (0.64-0.89)**	0.78 (0.66-0.92)**	0.80 (0.67-0.96)**	0.92 (0.69-1.22)
Not stated	3,286 (14.1%)	694 (17.4%)	0.74 (0.68-0.82)**	0.78 (0.71-0.86)**	0.79 (0.72-0.87)**	0.79 (0.72-0.87)**
**Resident within Local catchment area**	18,100 (77.8%)	4,064 (72.1%)	0.74 (0.69-0.76)**	0.88 (0.82-0.95)**	0.89 (0.83-0.96)**	0.87 (0.80-0.94)**
**National quartiles of Neighbourhood deprivation**						
1st (Most deprived)	11,326 (79.7%)	2,884 (51.1%)	Reference	Reference	Reference	Reference
2nd	7,891 (33.9%)	1,785 (31.7%)	0.89 (0.83-0.94)**	0.83(0.76-0.89)**	0.82 (0.77-0.88)**	0.85 (0.79-0.92)**
3rd	2,349 (10.1%)	557 (9.9%)	0.93 (0.84-1.03)	0.74(0.69-0.83)**	0.74 (0.66-0.83)**	0.78 (0.69-0.89)**
4th (Least Deprived)	1,692(7.3%)	413 (7.3%)	0.96 (0.85-1.07)	0.70(0.62-0.80)**	0.70 (0.62-0.80)**	0.69 (0.62-0.78)**
**Address data available[4]**						
Postcode 1	14,119(60.7%)	3,170 (56.2%)	Reference	Reference	Reference	Reference
Postcode 2	2,287 (9.8%)	669 (11.9%)	1.30 (1.18-1.43)**	0.71(0.63-0.78)**	0.71 (0.64-0.81)**	0.85 (0.65-1.11)
Postcode 3	4,618 (19.9%)	1,077 (19.1%)	1.03 (0.96-1.12)**	0.92(0.84-0.99)*	0.92 (0.85-1.00)	1.01 (0.87-1.19)
Postcode 4	1,448 (6.2%)	485 (8.6%)	1.49 (1.33-1.67)**	0.81(0.71-0.93)**	0.82 (0.72-0.95)**	1.14 (0.71-1.81)
Postcode 5	788 (3.4%)	238 (4.2%)	1.34 (1.16-1.56)**	0.93(0.79-1.10)	0.93 (0.78-1.09)	1.85 (0.74-4.66)

*P<0.05,**P<0.01, [1]adjusted for all other co-variates listed in the table. [2] adjusted model with inverse probability weighting for matching included, [3]adjusted model with addition of matching probability estimates entered as a co-variate, [4] See Figure 1 legend

For peer review only - http://bmjopen.bmj.com/site/about/guidelines.xhtml

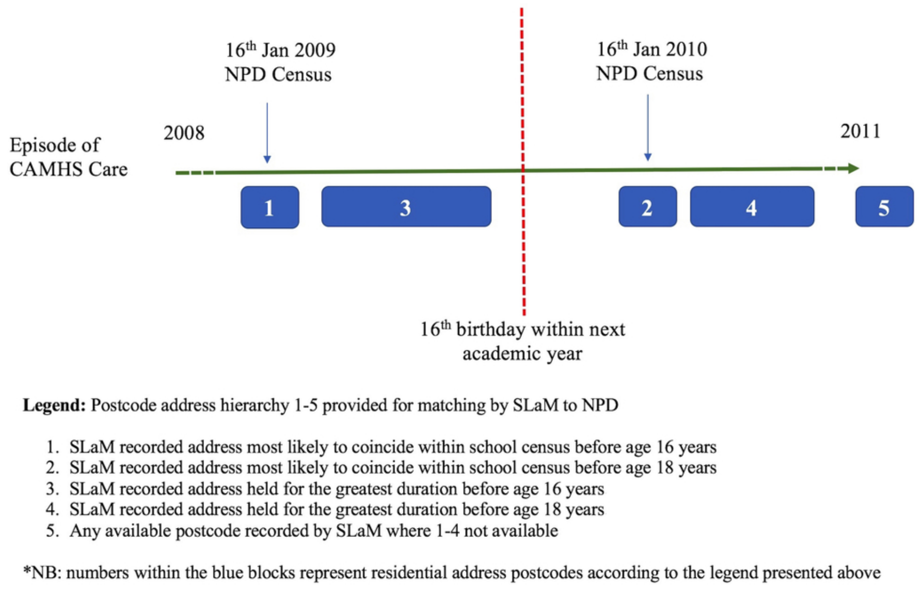

Figure 1: Creating a hierarchy of matching postcodes* to improve the link between CRIS CAMHS Data to DfE National Pupil Database

90x55mm (300 x 300 DPI)

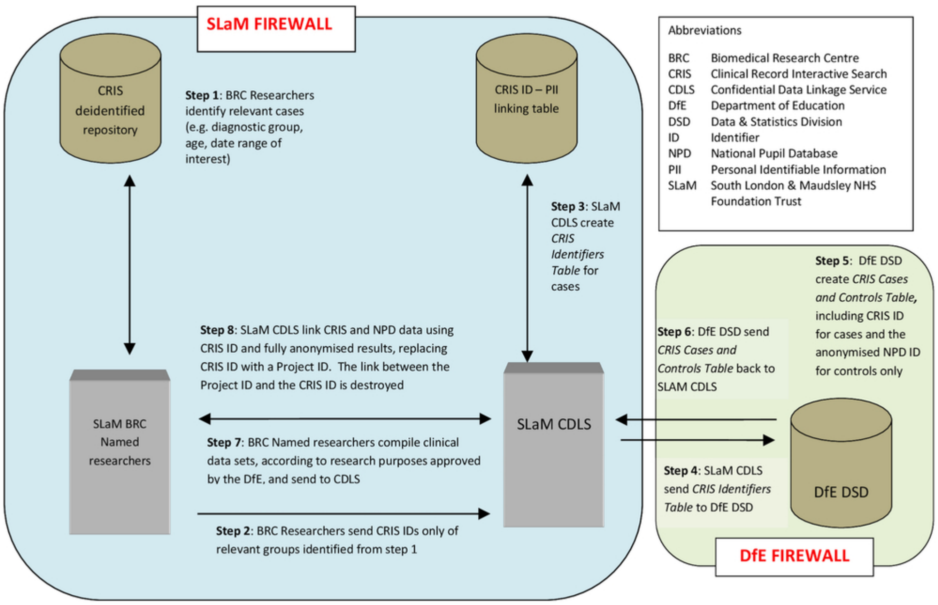

Figure 2: Data flow process linking CRIS CAMHS Data to the National Pupil Database

90x63mm (300 x 300 DPI)

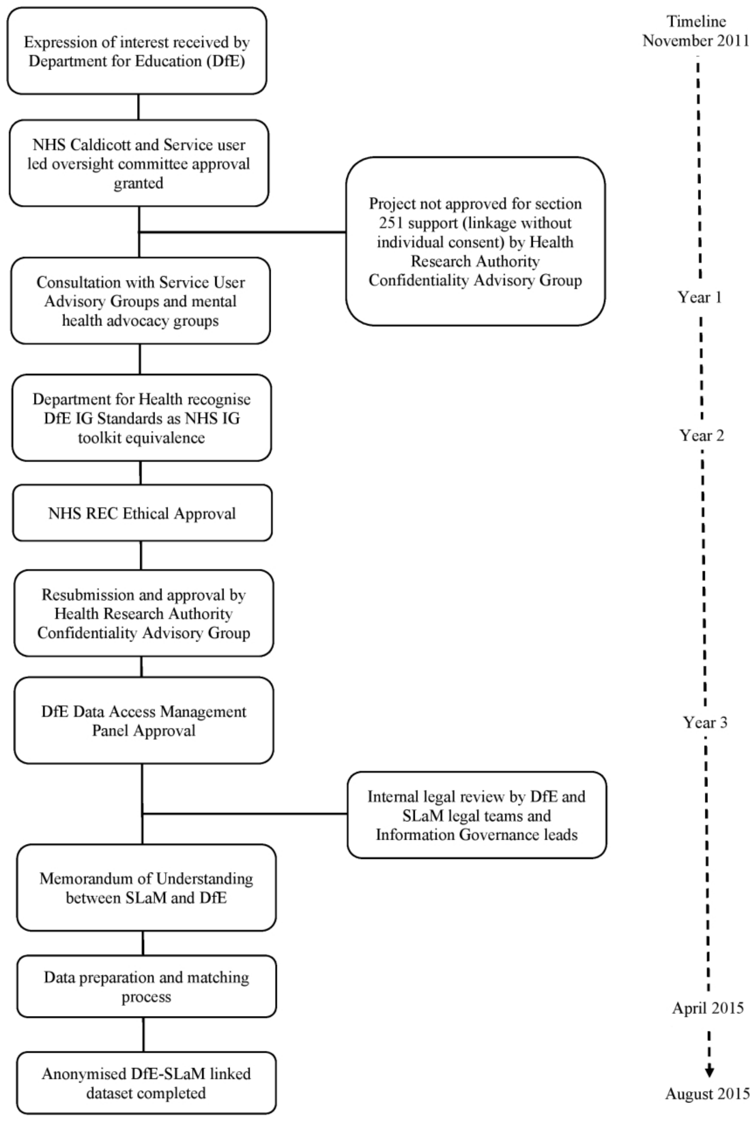

Figure 3: A timeline of the ethical, legal and technical milestones for reaching a data linkage between DfE and SLaM

210x280mm (300 x 300 DPI)

SUPPLEMENTARY MATERIAL

Title: An approach to linking education, social care and electronic health records for children and young people attending mental health services

Johnny Downs, Tamsin Ford, Robert Stewart, Hitesh Shetty, Ryan Little, Amelia Jewell, Matthew Broadbent, Jessica Deighton, Tarek Mostafa, Ruth Gilbert, Matthew Hotopf and Richard Hayes

* Corresponding author contact information:

Dr Johnny Downs, Department of Child and Adolescent Psychiatry, IOPPN Biomedical Research Centre Nucleus, Ground Floor Mapother House PO BOX 92, King's College London, De Crespigny Park, Denmark Hill, London SE5 8AF. Tel: +44 (0)20 3228 8553. Email: johnny.downs@kcl.ac.uk

Supplementary report on providing a linked health and educational data resource: achieving the ethical, governance and legal approvals

Initiating the discussion on the purpose and process of the linkage between public sector data controllers

We first approached the Department for Education directly who held nationally collected education data via termly school submissions to the National Pupil Database.[1] We planned a linkage with national data, as opposed to regional data sources held by the local education authorities, to prevent clinical sample attrition. We expected a considerable proportion of children and young people receiving SLaM treatment would reside outside the SLaM Catchment area or potentially move outside the catchment after treatment. In addition, the Department for Education had relatively transparent systems, and a dedicated office, for managing requests for educational data extracts, through their National Pupil Database Team. Once Research Governance approval was granted by the SLaM Caldicott Guardian Committee and the DfE's Data Management Advisory Panel, we prepared an application to the Health Research Authority Confidentiality Advisory Group (HRA CAG).[2] The HRA CAG have the authority to provide recommendations on behalf of the Secretary of State for Health to permit the linkage of NHS data without individual patient consent for the purposes of research, if it meets the criteria within section 251 of the NHS Act 2006. The main purpose of our application was to examine and

For peer review only - http://bmjopen.bmj.com/site/about/guidelines.xhtml

estimate the effects of clinically recognised, mental health disorder and treatment on educational outcomes.

The HRA CAG rejected the first application, as the research activity proposed did not demonstrate sufficient medical purpose and public benefit to meet the s251 requirements. It was highlighted by the HRA CAG that support under current regulations could only be provided where potential public benefit were sufficiently defined.[3] In particular, it was noted that in order to satisfy one of the conditions in schedule 3 of the Data Protection Act (32) (required to process sensitive personal data including data relating to an individual's physical or mental health) a medical purpose would also need to be specified; education outcomes in themselves would not suffice as a medical outcome. A second issue, was the lack of consideration of a practicable alternative to the use of confidential patient information without consent.

The HRA CAG also queried whether we had considered if the Health and Social Care Information Centre (HSCIC, now NHS Digital)[4] could carry out the linkages on the applicant's behalf using their Trusted Data Linkage Service. The CAG advised that this route would negate the requirement for SLaM to disclose confidential patient information to the DfE, and minimise the disclosure of patient information. A final major issue related to the governance arrangements in place around the processing of patient data by the DfE. We hadn't provided sufficient information around retention periods, access arrangements and the extent of identifiable data requested.

Defining 'medical purpose' and public benefit when seeking s251 support

To prepare for resubmission, we examined the issues identified by the HRA CAG. The initial application took a broad interpretation of 'medical purpose.' Given our clinical experience working in CAMHS, and the time CAMHS devoted to improving children and young people's function in school, we had presumed that educational outcomes for those with psychiatric diagnosis were salient to 'a medical purpose.' As a result, we underestimated the need to demonstrate to the CAG that educational performance (attainment, attendances and exclusions) were viewed by researchers, and NHS clinicians working within CAMHS, as key medical outcomes. Also, we had not made a clear enough case for using the linked educational data to examine the aetiological factors for child onset psychiatric disorders. These issues were addressed in the revised scientific proposal, largely by describing research that would examine the bi-directional associations between educational performance and mental health disorders.

For peer review only

In terms of gathering evidence for support of the public benefit to use patient identifiable data via CRIS to link to the national pupil database without patient or caregiver consent, we consulted several clinical, patient and caregiver groups. We gave presentations and collected minutes from the SLaM child and adolescent psychiatry executive group, the Service User Research Enterprise group (SURE), the service user led CRIS Oversight Committee, and SLaM-involved parents, through the BRC patient engagement programme.[5] Because of the focus of one of the projects using the linked data was an investigation into the educational outcomes of children and young people with Autism Spectrum Disorders, we also invited comments on the proposal from the National Autistic Society.

Identifying a trusted third party for managing health data linkages

To address the second issue, we provided an overview to the CAG of the advantages and disadvantages of using NHS Digital as a trusted third party to conduct linkages between SLaM and NPD data. We acknowledged that using NHS Digital would not require SLaM to release patient identifiers of over 35,500 names and addresses to the DfE. However, we described this advantage as fairly limited. We argued that the method proposed would involve no release of clinical data to the DfE, and that mental health status data were already collected and available to informaticians working in DfE National Pupil Database Team under their Special Education Need fields. In addition, we explained that DfE informaticians were already contracted to work with highly sensitive information at an individual level (for example, child protection status, benefit status of parents etc.) under comparable data governance standards expected of NHS Digital informaticians, as detailed by HMG Security Policy Framework v10 2013 (SPF).[6] We acknowledged that an additional potential benefit to using NHS Digital was that patient identifiers would be retained within a NHS environment. But after we invited Department of Health (DoH) and DfE to discuss Information Governance standards between their respective departments (in this case HSCIC and DfE Data Division) they advised, and the data controllers accepted, that there was little difference in data security policy. The DoH official responsible for NHS Digital Information Security and Risk Management Policy liaised with the DfE Departmental Security Unit Information Assurance Policy & Governance Team Leader, and reviewed the DfE Data and Statistics Division internal data processing, information handling controls, and assurance regimes. DoH confirmed that the DfE were in line with government standards and meet equivalent to IG expectations for NHS care system organisations.[7]

To provide further argument for not using NHS Digital as the trusted third party in this linkage, we described two alternatives routes, where NHS Digital performed the linkage and avoided transfer of NHS identifiers to the DfE. One route involved NHS Digital receiving all 15 million identifiers from the DfE, conducting the complex matching with the SLaM identifiers, completing the anonymisation process, and then providing a pseudo-anonymised dataset to SLaM. The second route involved NHS Digital receiving 15 million identifiers from the DfE, conducting the matching process, sending SLaM the controls and cases table with matched SLaM & NPD pseudonyms, and then sending controls and cases with just NPD pseudonym (the DfE remain blinded to SLaM case status) back to the DfE. After this, the DfE would then have to match the education variables of interest on the NPD pseudonym to create a pseudo-anonymised NPD variables table, and finally, send the pseudo-anonymised NPD variables to the SLaM CDLS for later matching with CRIS data. Both SLaM and DfE data controllers were concerned with the number of identifiers that would need to be transferred in both these processes, with sensitive educational variables being conveyed twice between the parties (DfE to NHS Digital, NHS Digital to SLaM CDLS). In addition, for both options DfE would need to supply identifiers for over 15 million individuals to NHS Digital, which may have contained a number of different addresses for each individual, and then separately convey over 500 education variables per individual, linked by pseudonym to the identifiers. DfE and SLaM data controllers, both expressed concerned that the harm caused to individuals if a breach of data security occurred in either of these processes could be significant, especially given the scale and sensitivity of the educational data, and the very large number of individuals involved. Hence, we advised the HRA CAG that both data controllers preferred to pursue a simpler linkage method, using the DfE to undertake the linkage of identifiers, within their secure environment and with appropriate governance controls using the minimum number of identifiers required.

Equivalence in data security requirements between health and education systems

This third issue was largely addressed by demonstrating data security equivalence between the DfE and DoH standards in processing and storing the data. In the re-submission to the CAG we confirmed that all personal identifiers were destroyed immediately after linkage and validation by the DfE, and that data was to be anonymised and only analysed within the same secure environment. The table linking NPD and CRIS pseudonyms, would be destroyed after 60 days from SLaM CDLS receiving the data, to permit some additional data cleaning and validation checks. With these additional details, the application was re-submitted and approved (ref CAG 9-08(a)/2013 0048).[8]

For peer review only - http://bmjopen.bmj.com/site/about/guidelines.xhtml

Completing the Memorandum of Understanding between Data Controllers

It took some time to formalise a Memorandum of Understanding (MoU) between the DfE and SLaM. This was due to it being the first time an NHS trust in England had entered into a data sharing contract with the DfE, and the lawyers representing both parties took time to become familiar with the legal basis for sharing data in the proposed manner. After a year under legal review, a signed agreement was eventually completed. One of the areas of contention regarded cross-indemnity. Standard legal advice for commercial data sharing often stipulate that each party should indemnify, and keep indemnified the other party, against any claims brought against them despite the proper performance of the Data Activities as envisaged by the MoU. So, taking this linkage project as an example, if someone were to legally challenge SLaM for data that related to the DfE, which they held temporarily during the matching process, then SLaM would honour an agreement to respond the challenge, and vice versa with the DfE. However, if responsibility was shared between parties, it could have potentially created problems in terms of interpretation, especially in relation to data protection compliance, especially for tasks that are time sensitive such as responding to subject access requests. We eventually reached an agreement that the parties would self–indemnify. This decision was aided by the data flows which provided a clear demarcation between DfE and SLaM data systems and procedures, which we came to understand was important when undertaking data processes on behalf of the other data controller. As SLaM and DfE responsibilities for the project were well defined, both agreed that if one party failed in its obligations, it was most likely that enforcement action would be carried out against the party that was in breach of their agreed obligations at that point in the linkage process.

For peer review only

REFERENCES

1 Department for Education. National Pupil Database User Guide. UK Government, 2015 https://www.gov.uk/government/uploads/system/uploads/attachment_data/file/261189/NPD_User_Guide.pdf (accessed May 3, 2018).
2 Health Research Authority. Section 251 and the Confidentiality Advisory Group (CAG). Health Res. Auth. http://www.hra.nhs.uk/about-the-hra/our-committees/section-251/ (accessed May 22, 2018).
3 Health Research Authority. Principles of Advice: Exploring the concepts of public interest and reseasonably practicable. .
4 NHS Digital. https://digital.nhs.uk/home (accessed May 20, 2018).
5 King's College London - Service User Research Enterprise (SURE). https://www.kcl.ac.uk/ioppn/depts/hspr/research/ciemh/sure/SURE.aspx (accessed May 22, 2018).
6 HMG Cabinet Office. Security policy framework. 2016. https://www.gov.uk/government/publications/security-policy-framework (accessed April 8, 2018).
7 Department for Health. NHS Information Governance Toolkit. https://www.igt.hscic.gov.uk/ (accessed April 8, 2018).
8 Health Research Authority. CAG Advice and HRA/SofS Approval Decisions. Health Res. Auth. https://www.hra.nhs.uk/planning-and-improving-research/application-summaries/confidentiality-advisory-group-registers/ (accessed May 22, 2018).

For peer review only

Supplementary Figure 1: Number of accepted first referrals for all children and young people (aged 4 -17) seen by SLaM CAMHS services (Sept 2007 – August 2013)

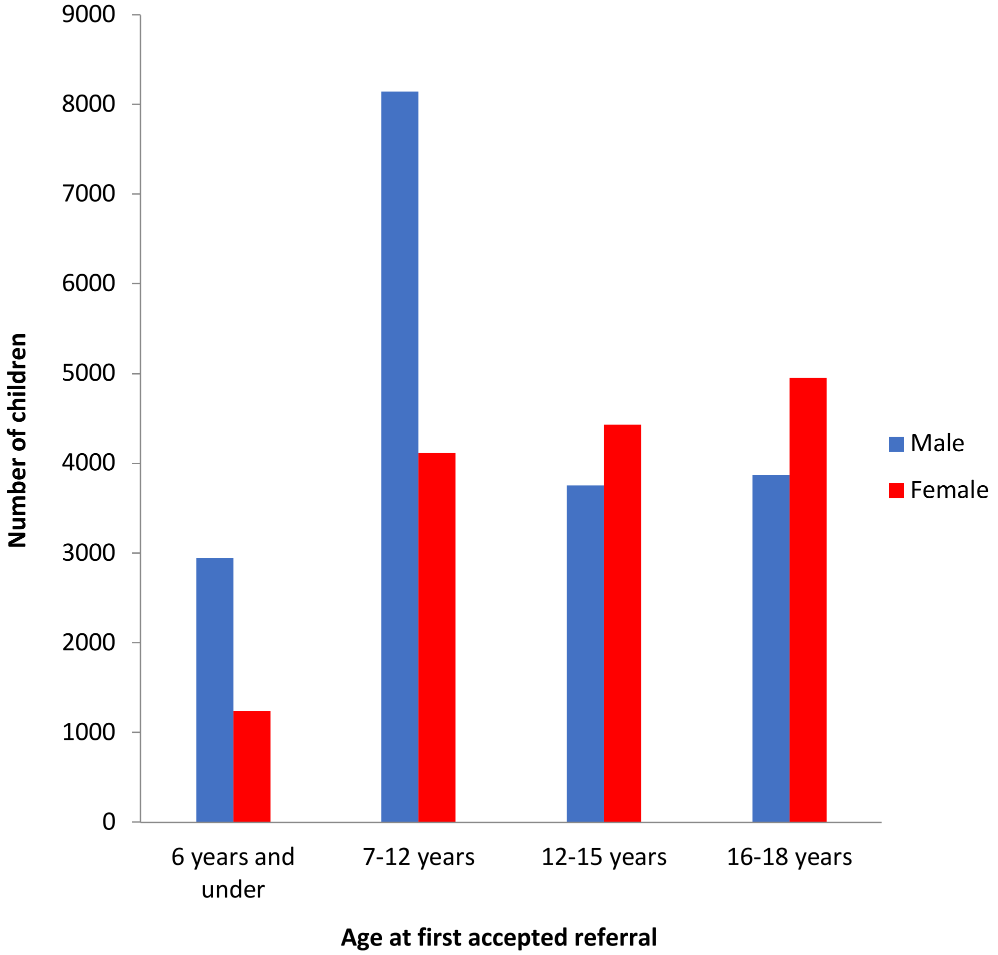

Supplementary Figure 2: Duration between first and last contact with mental health professionals for children and young people (aged 4 -17) accepted to SLAM CAMHS between Sept 2007 – August 2013.

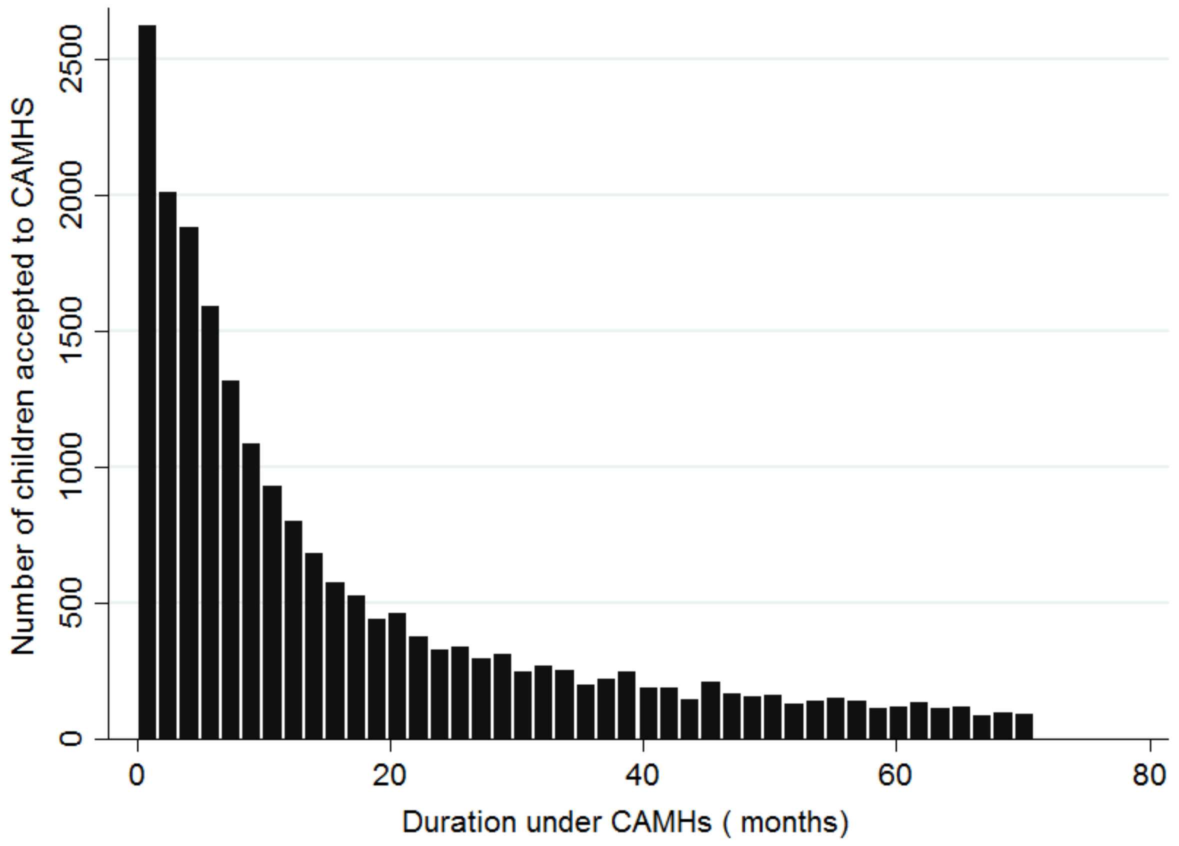

Supplementary table 1: Diagnostic breakdown of all children (aged 4 -17) referred to SLaM CAMHS services between Sept 2007 and August 2013.

		Local Catchment Area*		National Catchment Area*	
ICD-10 Psychiatric Diagnostic Classification		Male (n=15204)	Female (n=11469)	Male (n=4522)	Female (n=4314)
		n (%)	n (%)	n (%)	n (%)
	Any ICD-10 Diagnosis	9315 (61.3)	6587 (57.4)	2592 (57.3)	2545 (59)
Axis One	Pervasive Developmental Disorders (F84)	2116 (13.9)	519 (4.5)	749 (16.5)	248 (5.9)
	Hyperkinetic Disorders (F90)	2345 (15.4)	435 (3.8)	801 (17.7)	210 (4.9)
	Conduct Disorders (F91)	2160 (14.2)	983 (8.6)	392 (8.7)	169 (3.9)
	Disorders due to psychoactive substance use (F10–F19)	253 (1.7)	180 (1.6)	112 (2.5)	53 (1.2)
	Psychotic Disorders (F20-F29, F30-F31, F32.3)	437 (2.9)	438 (3.8)	239 (5.3)	239 (5.5)
	Depression and other (affective) disorders (F32–F39)	733 (4.8)	1497 (13.1)	197 (4.4)	511 (11.8)
	Emotional and stress related disorders (F40-F48, F93, F94, F98)	2442 (16.1)	2930 (25.5)	522 (11.5)	879 (26.4)
	Post-Traumatic Stress Disorder (F43)	269 (1.8)	330 (2.9)	64 (1.4)	105 (2.7)
	Obsessive Compulsive Disorder (F42)	201 (1.3)	220 (1.9)	269(5.9)	164 (3.9)
	No recorded Axis One Diagnosis	5889 (38.7)	4882 (42.6)	1929 (42.7)	1770 (41.0)
Axis Two	Disorders of Scholastic Development (F80-F89)	1048 (6.9)	337 (2.9)	195 (4.3)	89 (3.1)
Axis Three	Intellectual Disorders (F70-F79)	870 (5.7)	357 (3.1)	443 (9.7)	195 (3.8)

*Note: The sample are split by residence, either within 4 London Boroughs served by local SLaM services (Local Catchment area), or from rest of England served by SLaM National and Specialist services (National Catchment Area)

For peer review only - http://bmjopen.bmj.com/site/about/guidelines.xhtml

The RECORD statement – checklist of items, extended from the STROBE statement, that should be reported in observational studies using routinely collected health data.

	Item No.	STROBE items	Location in manuscript where items are reported	RECORD items	Location in manuscript where items are reported
**Title and abstract**					
	1	(a) Indicate the study's design with a commonly used term in the title or the abstract (b) Provide in the abstract an informative and balanced summary of what was done and what was found		RECORD 1.1: The type of data used should be specified in the title or abstract. When possible, the name of the databases used should be included.<br><br>RECORD 1.2: If applicable, the geographic region and timeframe within which the study took place should be reported in the title or abstract.<br><br>RECORD 1.3: If linkage between databases was conducted for the study, this should be clearly stated in the title or abstract.	**Yes stated in the title and abstract – page 3**
**Introduction**					
Background rationale	2	Explain the scientific background and rationale for the investigation being reported			Yes, stated in the pages 4 & 5 within the introduction section
Objectives	3	State specific objectives, including any prespecified hypotheses			Pages 5 within the introduction section
**Methods**					
Study Design	4	Present key elements of study design early in the paper			**Yes, provided in the methods section pages 6-9**
Setting	5	Describe the setting, locations,			

For peer review only

		and relevant dates, including periods of recruitment, exposure, follow-up, and data collection			
Participants	6	*(a) Cohort study* - Give the eligibility criteria, and the sources and methods of selection of participants. Describe methods of follow-up<br>*Case-control study* - Give the eligibility criteria, and the sources and methods of case ascertainment and control selection. Give the rationale for the choice of cases and controls<br>*Cross-sectional study* - Give the eligibility criteria, and the sources and methods of selection of participants<br><br>*(b) Cohort study* - For matched studies, give matching criteria and number of exposed and unexposed<br>*Case-control study* - For matched studies, give matching criteria and the number of controls per case		RECORD 6.1: The methods of study population selection (such as codes or algorithms used to identify subjects) should be listed in detail. If this is not possible, an explanation should be provided.<br><br>RECORD 6.2: Any validation studies of the codes or algorithms used to select the population should be referenced. If validation was conducted for this study and not published elsewhere, detailed methods and results should be provided.<br><br>RECORD 6.3: If the study involved linkage of databases, consider use of a flow diagram or other graphical display to demonstrate the data linkage process, including the number of individuals with linked data at each stage.	**6.1 Yes, provided in the methods section pages 6-9**<br><br>**6.2 Yes, provided in the methods section pages 6-9**<br><br>**6.3 Yes, provided page 9 of the methods, results page 12 and in figures 2 and 3**
Variables	7	Clearly define all outcomes, exposures, predictors, potential confounders, and effect modifiers. Give diagnostic criteria, if applicable.		RECORD 7.1: A complete list of codes and algorithms used to classify exposures, outcomes, confounders, and effect modifiers should be provided. If these cannot be reported, an explanation should be provided.	**Yes, provided in the methods section pages 6-9**
Data sources/ measurement	8	For each variable of interest, give sources of data and details of methods of assessment (measurement).<br>Describe comparability of			**Yes, provided in the methods section pages 6-9**

For peer review only

		assessment methods if there is more than one group			
Bias	9	Describe any efforts to address potential sources of bias			**Yes, provided in the methods (statistical analysis section pg 10-11)**
Study size	10	Explain how the study size was arrived at			**Yes, provided in the methods section pages 6-9**
Quantitative variables	11	Explain how quantitative variables were handled in the analyses. If applicable, describe which groupings were chosen, and why			**Yes, provided in the methods section pages 6-9**
Statistical methods	12	(a) Describe all statistical methods, including those used to control for confounding<br>(b) Describe any methods used to examine subgroups and interactions<br>(c) Explain how missing data were addressed<br>(d) *Cohort study* - If applicable, explain how loss to follow-up was addressed<br>*Case-control study* - If applicable, explain how matching of cases and controls was addressed<br>*Cross-sectional study* - If applicable, describe analytical methods taking account of sampling strategy<br>(e) Describe any sensitivity analyses			**Yes, provided in the methods (statistical analysis section pg 10-11)**

For peer review only

Data access and cleaning methods		..		RECORD 12.1: Authors should describe the extent to which the investigators had access to the database population used to create the study population.<br><br>RECORD 12.2: Authors should provide information on the data cleaning methods used in the study.	**Yes, provided in the methods section pages 6-9**
Linkage		..		RECORD 12.3: State whether the study included person-level, institutional-level, or other data linkage across two or more databases. The methods of linkage and methods of linkage quality evaluation should be provided.	**Yes, main focus of the results page 12-13**
**Results**					
Participants	13	(a) Report the numbers of individuals at each stage of the study (*e.g.*, numbers potentially eligible, examined for eligibility, confirmed eligible, included in the study, completing follow-up, and analysed)<br>(b) Give reasons for non-participation at each stage.<br>(c) Consider use of a flow diagram		RECORD 13.1: Describe in detail the selection of the persons included in the study (*i.e.,* study population selection) including filtering based on data quality, data availability and linkage. The selection of included persons can be described in the text and/or by means of the study flow diagram.	**Yes, provided in the results page 12-13, Figures 1-3, and tables 1 and 2**
Descriptive data	14	(a) Give characteristics of study participants (*e.g.*, demographic, clinical, social) and information on exposures and potential confounders<br>(b) Indicate the number of participants with missing data for each variable of interest<br>(c) *Cohort study* - summarise follow-up time (*e.g.*, average and total amount)			**Yes, provided in the results page 12-13, Figures 1-3, and tables 1 and 2**

For peer review only

Outcome data	15	*Cohort study* - Report numbers of outcome events or summary measures over time<br>*Case-control study* - Report numbers in each exposure category, or summary measures of exposure<br>*Cross-sectional study* - Report numbers of outcome events or summary measures			**Yes, provided in the results page 12-13, tables 1 and 2**
Main results	16	(a) Give unadjusted estimates and, if applicable, confounder-adjusted estimates and their precision (e.g., 95% confidence interval). Make clear which confounders were adjusted for and why they were included<br>(b) Report category boundaries when continuous variables were categorized<br>(c) If relevant, consider translating estimates of relative risk into absolute risk for a meaningful time period			**Yes, provided in the results page 12-13, tables 1 and 2**
Other analyses	17	Report other analyses done—e.g., analyses of subgroups and interactions, and sensitivity analyses			**Yes, provided in the results page 12-13, tables 1 and 2**
**Discussion**					
Key results	18	Summarise key results with reference to study objectives			
Limitations	19	Discuss limitations of the study, taking into account sources of potential bias or imprecision. Discuss both direction and magnitude of any potential bias		RECORD 19.1: Discuss the implications of using data that were not created or collected to answer the specific research question(s). Include discussion of misclassification bias, unmeasured confounding, missing data, and changing eligibility over time, as	**Yes, provided in the discussion, limitations section page 17**

For peer review only

				they pertain to the study being reported.	
Interpretation	20	Give a cautious overall interpretation of results considering objectives, limitations, multiplicity of analyses, results from similar studies, and other relevant evidence			**Yes, provided in the discussion, limitations section page 17**
Generalisability	21	Discuss the generalisability (external validity) of the study results			**Yes, provided in the discussion, section page 14**
**Other Information**					
Funding	22	Give the source of funding and the role of the funders for the present study and, if applicable, for the original study on which the present article is based			**Yes, funders statement has been included [page 19]**
Accessibility of protocol, raw data, and programming code		..		RECORD 22.1: Authors should provide information on how to access any supplemental information such as the study protocol, raw data, or programming code.	**Online Supplementary material referenced in text, owing to governance restrictions raw data not available but contact details provided for specific queries / replication work**

*Reference: Benchimol EI, Smeeth L, Guttmann A, Harron K, Moher D, Petersen I, Sørensen HT, von Elm E, Langan SM, the RECORD Working Committee. The REporting of studies Conducted using Observational Routinely-collected health Data (RECORD) Statement. *PLoS Medicine* 2015; in press.

*Checklist is protected under Creative Commons Attribution (CC BY) license.

For peer review only
